# Single-cell spatial landscapes of the lung tumour immune microenvironment

**DOI:** 10.1038/s41586-022-05672-3

**Published:** 2023-02-01

**Authors:** Mark Sorin, Morteza Rezanejad, Elham Karimi, Benoit Fiset, Lysanne Desharnais, Lucas J. M. Perus, Simon Milette, Miranda W. Yu, Sarah M. Maritan, Samuel Doré, Émilie Pichette, William Enlow, Andréanne Gagné, Yuhong Wei, Michele Orain, Venkata S. K. Manem, Roni Rayes, Peter M. Siegel, Sophie Camilleri-Broët, Pierre Olivier Fiset, Patrice Desmeules, Jonathan D. Spicer, Daniela F. Quail, Philippe Joubert, Logan A. Walsh

**Affiliations:** 1 Rosalind and Morris Goodman Cancer Institute, McGill University, Montreal, Quebec Canada; 2 Department of Human Genetics, McGill University, Montreal, Quebec Canada; 3 Department of Psychology, University of Toronto, Toronto, Ontario Canada; 4 Department of Computer Science, University of Toronto, Toronto, Ontario Canada; 5 Department of Physiology, Faculty of Medicine, McGill University, Montreal, Quebec Canada; 6 Department of Medicine, Division of Experimental Medicine, McGill University, Montreal, Quebec Canada; 7 Faculty of Medicine and Health Sciences, McGill University, Montreal, Quebec Canada; 8 Institut Universitaire de Cardiologie et de Pneumologie de Québec, Laval University, Québec City, Quebec Canada; 9 Department of Mathematics and Computer Science, University of Quebec at Trois-Rivières, Trois-Rivières, Quebec Canada; 10 Department of Biochemistry, Faculty of Medicine, McGill University, Montreal, Quebec Canada; 11 Department of Pathology, McGill University, Montreal, Quebec Canada; 12 Department of Surgery, McGill University Health Center, Montreal, Quebec Canada

**Keywords:** Cancer microenvironment, Non-small-cell lung cancer

## Abstract

Single-cell technologies have revealed the complexity of the tumour immune microenvironment with unparalleled resolution^1–9^. Most clinical strategies rely on histopathological stratification of tumour subtypes, yet the spatial context of single-cell phenotypes within these stratified subgroups is poorly understood. Here we apply imaging mass cytometry to characterize the tumour and immunological landscape of samples from 416 patients with lung adenocarcinoma across five histological patterns. We resolve more than 1.6 million cells, enabling spatial analysis of immune lineages and activation states with distinct clinical correlates, including survival. Using deep learning, we can predict with high accuracy those patients who will progress after surgery using a single 1-mm^2^ tumour core, which could be informative for clinical management following surgical resection. Our dataset represents a valuable resource for the non-small cell lung cancer research community and exemplifies the utility of spatial resolution within single-cell analyses. This study also highlights how artificial intelligence can improve our understanding of microenvironmental features that underlie cancer progression and may influence future clinical practice.

## Abstract (web summary)

Using imaging mass cytometry, the tumour and immunological spatial landscapes of 416 lung adenocarcinomas are characterized, which, when combined with deep learning, can predict clinical outcomes with high accuracy.

## Main

Lung cancer remains the leading cause of cancer-related death, accounting for greater than 20% of all cancer mortalities^10^. Lung adenocarcinoma (LUAD), a type of non-small cell lung cancer (NSCLC), is the most common subtype and is characterized by distinct cellular and molecular features^11^. The tumour immune microenvironment (TIME) is a major source of LUAD heterogeneity and influences both disease progression and response to therapy^1,3,5^. The positioning of immune cells within tumours is known to dictate their function^12–14^; therefore, understanding the spatial landscape of the lung TIME would provide mechanistic insights into disease progression, reveal novel therapeutic vulnerabilities and unveil biomarkers of response to existing treatments. Here, using highly multiplexed imaging mass cytometry (IMC), we interrogated spatially resolved features of the TIME that are associated with clinical outcomes in patients with LUAD. Using a deep neural network model, we demonstrated that various clinical outcomes, such as progression, can be predicted using features that an artificial intelligence-based system can extract from raw IMC images. The ability to identify patients who will progress with a high degree of certainty could guide future post-surgical management.

## LUAD tumour immune microenvironment

**Fig. 1:**
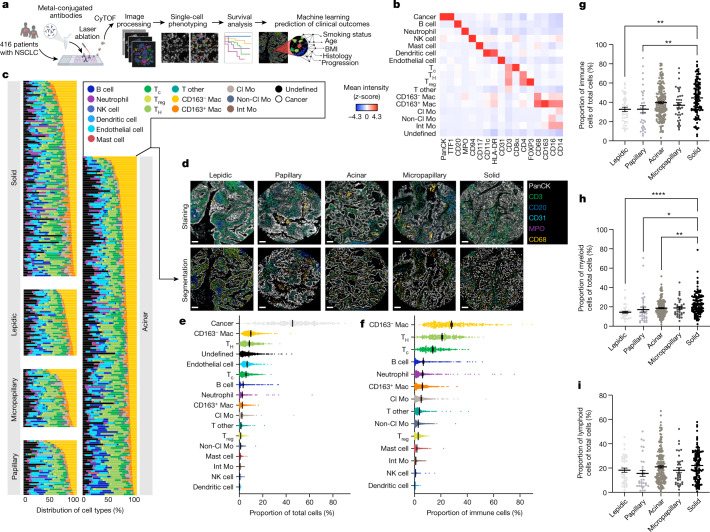
IMC defines the spatial landscape of LUAD. **a**, Schematic depicting IMC acquisition of multiplexed images from 416 patients with LUAD, single-cell phenotyping, survival and machine learning prediction of clinical outcomes. CyTOF, cytometry by time of flight. Images were created with BioRender. **b**, Average expression of lineage markers across cell types in the LUAD tissue using the panel of isotope-conjugated antibodies. Cl Mo, classical monocyte; Int Mo, intermediate monocyte; Mac, macrophage; NK, natural killer; non-Cl Mo, non-classical monocyte; T_c_, cytotoxic T cell; T_H_, helper T cell. **c**, Waterfall plot depicting the distribution of 16 stromal and immune cell types across histological subgroups. **d**, Representative images of antibody staining and corresponding single-cell segmented images across histological subgroups. Scale bars, 100 μm. **e**,**f**, Prevalence of 17 cell types, including 14 immune cell types, across 416 patients with LUAD as a proportion of total cells (**e**) and immune cells (**f**). **g**–**i**, Prevalence of all immune (**g**), myeloid (**h**) and lymphoid (**i**) cells across lepidic (*n* = 40), papillary (*n* = 33), acinar (*n* = 190), micropapillary (*n* = 35) and solid (*n* = 118) architectural patterns as a proportion of total cells. Comparison between lepidic and solid (immune cells): **P = 0.0013. Comparison between papillary and solid (immune cells): **P = 0.0039. Comparison between lepidic and solid (myeloid cells): ****P ≤ 0.0001. Comparison between papillary and solid (myeloid cells): *P = 0.0474. Comparison between acinar and solid (myeloid cells): **P = 0.0072. Data shown as mean ± s.e.m. (**e**–**i**). One-way ANOVA with Tukey multiple comparison test was used for statistical analysis (**g**–**i**).

**Extended Data Fig. 1:**
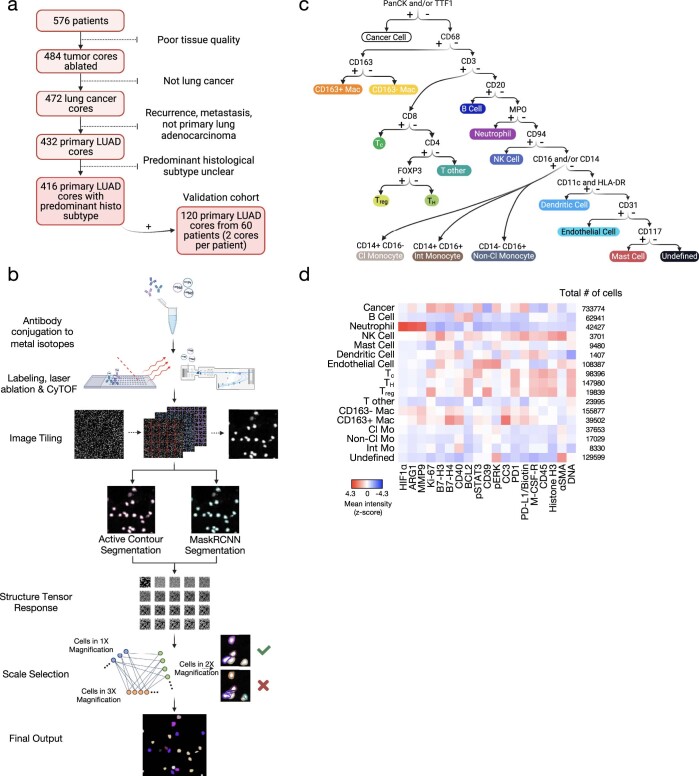
Imaging mass cytometry segmentation pipeline and antibody panel. **a**, Flowchart for inclusion and exclusion criteria of 576 lung adenocarcinoma cores. **b**, Schematic of IMC segmentation pipeline representing antibody conjugation of metal isotopes, labeling, laser ablation, CyTOF acquisition, image tiling, structure tensor response, scale selection and final output. **c**, Schematic depiction of the workflow and specific markers used for lineage assignment. Panels **a**–**c** were created with BioRender. **d**, Average expression of non-lineage markers across cell types in lung adenocarcinoma tissue stained with the panel of isotope-conjugated antibodies; CD163^−^Mac, CD163^−^ macrophage; CD163^+^ Mac, CD163^+^ macrophage; T_c_, CD8^+^ T cell; T_reg_, regulatory T cell, T_H_, CD4^+^ T cell; Cl Mo, classical monocyte; Int Mo, intermediate monocyte; Non-Cl Mo, non-classical monocyte; NK Cell, natural killer cell.

**Extended Data Fig. 2:**
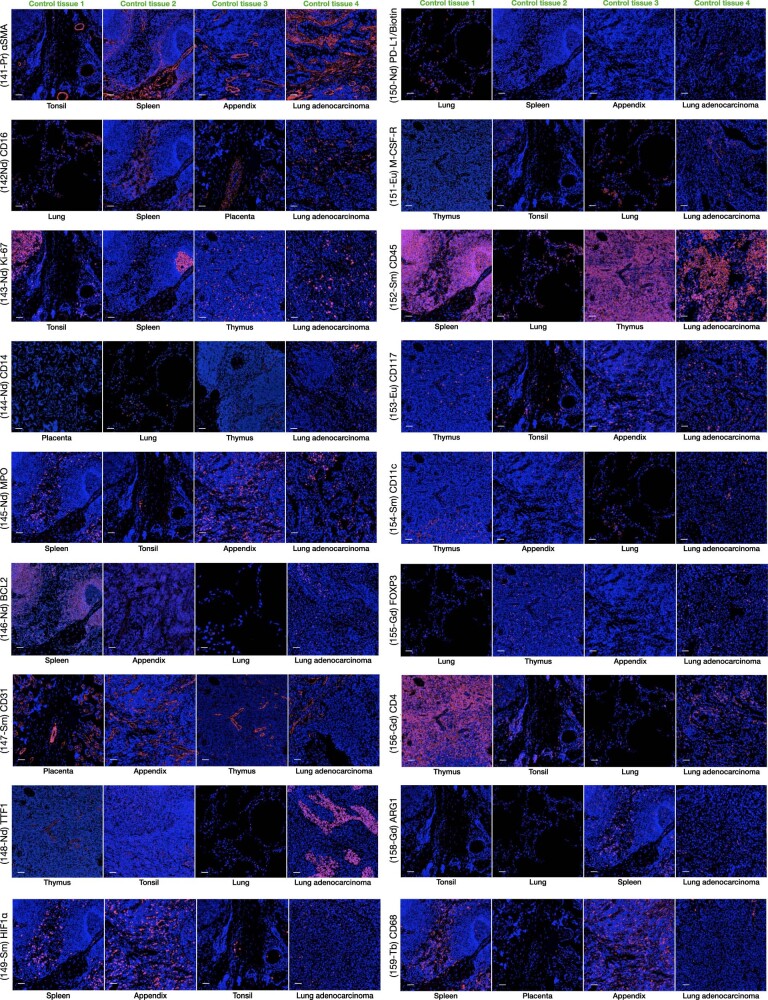
Antibody panel validation 1. Validation of 18 antibodies used for multiplex IMC across positive and negative controls. Scale bars represent 50 μm.

**Extended Data Fig. 3:**
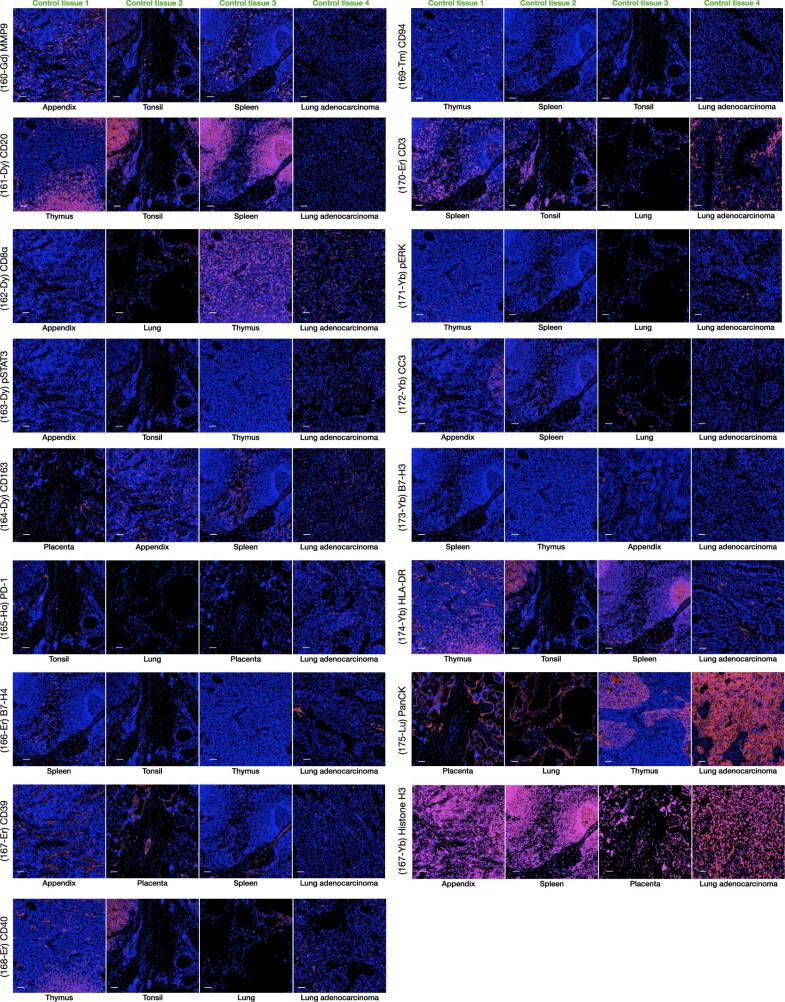
Antibody panel validation 2. Validation of 17 antibodies used for multiplex IMC across positive and negative controls. Scale bars represent 50 μm.

**Extended Data Fig. 4:**
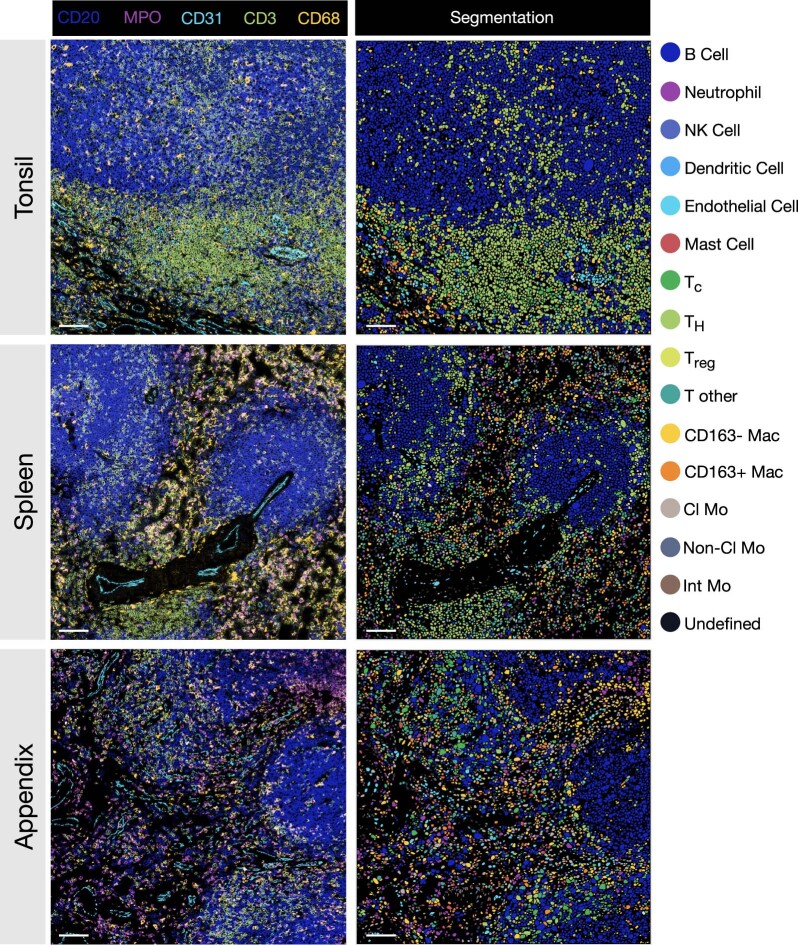
Antibody panel validation 3. Lineage marker staining and corresponding cell segmentation in control tissue. Colour code for cell segmentation is provided. Scale bars represent 100 μm.

**Extended Data Fig. 5:**
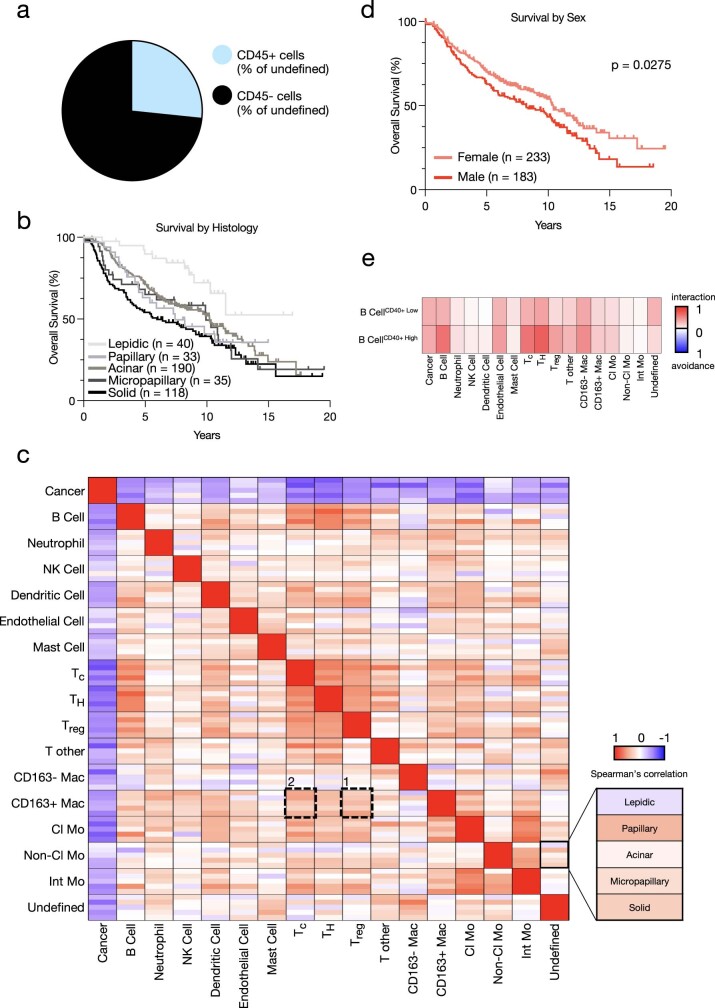
Survival analysis of distinct clinical variables. **a**, Pie chart depicting the proportion of undefined cells that are CD45^+^ across 416 lung adenocarcinoma patients. **b**, Kaplan–Meier curves of overall survival for 416 lung adenocarcinoma patients based on histological subgroup (Lepidic n = 40, Papillary n = 33, Acinar n = 190, Micropapillary n = 35, Solid n = 118. For P values, see Supplementary Table 3. **c**, Heatmap depicting the Spearman’s rank correlation coefficient with high coefficients in red and low coefficients in blue between indicated cell types across the five histological subgroups (Lepidic n = 40 images, Papillary n = 33 images, Acinar n = 190 images, Micropapillary n = 35 images, Solid n = 118 images). **d**, Kaplan–Meier curves of overall survival for 416 lung adenocarcinoma patients based on sex (Female n = 233, Male n = 183). **e**, Heatmap depicting cell-cell interaction/avoidance among B cells in the CD40^+^ B cell high (z score ≥0; %total B cells) and CD40^+^ B cell low (z score <0; %total B cells) groups. Each rectangle depicts significant pairwise cell-type interaction (red) or avoidance (blue) between indicated cell types (n = 186 images for the CD40^+^ B cell high group; n = 230 images for the CD40^+^ B cell low group; 1,000 permutations each). Statistical analysis (**b, d:** log-rank test).

To spatially characterize the cellular landscape of the lung TIME, we applied IMC to samples from 416 patients with LUAD (Fig. 1a, Extended Data Fig. 1a and Supplementary Table 1). We optimized a 35-plex antibody panel to identify cancer cells, stromal cells, and innate and adaptive immune lineages with diverse functional substates (Extended Data Figs. 1b–d and 2–4 and Supplementary Table 2). In total, we detected 1,644,178 cells and used a supervised lineage assignment approach to classify 14 distinct immune cell populations, along with tumour cells and endothelial cells using canonical lineage markers (Fig. 1b–f and Extended Data Figs. 1c and 5a).

Consistent with previous work^15^, high-grade solid tumours had the greatest immune infiltrate (44.6%) compared with micropapillary, acinar, papillary and lepidic architectures (37.0%, 39.7%, 32.8% and 32.7% respectively; Fig. 1g). This was driven by shifts within the myeloid compartment, as there were no significant differences in the average frequency of total lymphoid cells across histological patterns (Fig. 1h,i). In particular, macrophages were the most frequent cell population within the lung TIME, representing 12.3% of total cells (Fig. 1e) and 34.1% of immune cells (Fig. 1f), consistent with their critical role in the NSCLC niche^16^. We found the highest enrichment of CD163^+^ macrophages (putative ‘M2-like’ or protumorigenic) in solid tumours, which are one of the most aggressive architectures (Extended Data Fig. 5b, Supplementary Table 3, Supplementary Fig. 1, xii). The prevalence of CD163^+^ macrophages was strongly correlated with FOXP3^+^ immunoregulatory T cells (T_reg_ cells) in the solid pattern (Extended Data Fig. 5c, box 1). This relationship was much less pronounced in low-grade lepidic and papillary architectures, which had a strong correlation between CD163^+^ macrophages and cytotoxic CD8^+^ T cells (Extended Data Fig. 5c, box 2). These associations suggest a potential interplay between macrophage and T cell populations in the TIME across LUAD patterns. Of note, solid tumours were also enriched for additional myeloid components, including neutrophils, non-classical monocytes and intermediate monocytes (Supplementary Fig. 1, ii, xiv and xv). Similarly to macrophages, these myeloid populations all exhibit diverse functional states in NSCLC biology^4^ and exemplify the complex heterogeneity that exists in the lung TIME.

## LUAD multicellular spatial interactions

**Fig. 2:**
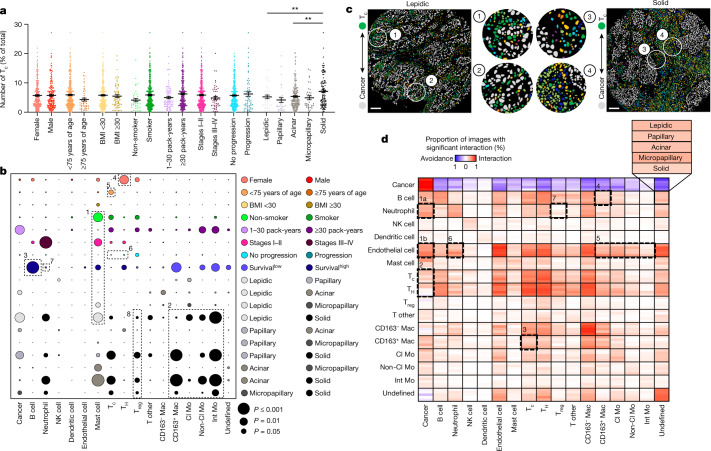
Variability in single-cell distributions across clinical variables and in cell–cell interaction profiles across histological patterns in LUAD. **a**, Prevalence of T_c_ cells (CD8^+^ T cells) across sex (female *n* = 233, male *n* = 183), age (younger than 75 years of age *n* = 369, 75 years of age or older *n* = 47), BMI (less than 30 *n* = 346, 30 or higher *n* = 70), smoking status (smoker *n* = 376, non-smoker *n* = 38), pack-years (1–30 *n* = 89, 30 or more *n* = 256), stage (I-II *n* = 365, III–IV *n* = 50), progression status (progression *n* = 64, no progression *n* = 340) and histological subgroup (lepidic *n* = 40, papillary *n* = 33, acinar *n* = 190, micropapillary *n* = 35, solid *n* = 118). Comparison between papillary and solid: **P = 0.0070, and acinar and solid: **P = 0.0076. Data shown as mean ± s.e.m. **b**, Bubble plot in which the circle size represents the level of significance and the circle colour indicates which of the two comparisons on the *y* axis has higher levels of the cell type on the *x* axis. Survival^low^, survival in the context of low (*z-*score < 0) cell prevalence. For *P* values, see Supplementary Table 4. **c**, Segmented images showing increased interaction of cancer and T_c_ cells in lepidic versus solid predominant LUAD. Scale bars, 100 μm. **d**, Heat map depicting significant pairwise cell–cell interaction (red) or avoidance (blue) across the five histological subgroups (lepidic *n* = 40 images, papillary *n* = 33 images, acinar *n* = 190 images, micropapillary *n* = 35 images, solid *n* = 118 images; 1,000 permutations each). The black boxes depict associations referenced in the text. FDR-corrected two-tailed Student’s *t*-test for sex, age, BMI, smoking status, pack-years, stage and progression status; one-way ANOVA with Tukey multiple comparison test for histological subgroup; and log-rank test for survival were used for statistical analysis (**a**,**b**).

We next assessed the relationship between immune populations and clinical or pathological variables by interrogating the frequency of individual cell types as a percentage of total cells within each image (Fig. 2a,b, Supplementary Tables 1 and 4 and Supplementary Fig. 1). Each image was cross-referenced with clinical data from patients, including sex, age, body mass index (BMI), smoking status, stage, progression, survival and histological subtype. Although we discovered established survival associations for several cell types^17,18^, most were driven by an enrichment in specific clinical or pathological groups. For example, although mast cells were associated with prolonged survival, they were overrepresented in non-smokers, early-stage patients and those with lepidic tumours (Fig. 2b, box 1)—all clinical variables associated with good outcomes. Similarly, CD163^+^ macrophages, non-classical monocytes and intermediate monocytes were enriched in solid tumours, which have poor outcomes (Fig. 2b, box 2). By contrast, B cell frequency was most significantly associated with better overall survival, independent of any confounding clinical or pathological variables (Fig. 2b, box 3).

Beyond survival associations, we found additional relationships between cell frequencies and specific clinical subgroups. For example, T cell subsets exhibited specific enrichment based on sex and age. Consistent with previous reports^19^, CD4^+^ helper T cells were significantly enriched in female patients (Fig. 2b, box 4), who have better overall survival than male patients^20,21^—an association also observed in our dataset (Extended Data Fig. 5d). Moreover, older patients (more than 75 years of age) had fewer intratumoural CD8^+^ T cells (Fig. 2b, box 5), reminiscent of immune ageing that is linked to reduced expression of co-stimulatory molecules, antigen receptor diversity and immunotherapy response^22–25^. Overall, these data reveal new relationships and add biological insight into established associations between cell frequencies and clinical outcomes within the TIME.

To gain insight into the cellular architecture and spatial organization of the LUAD TIME, we characterized direct interactions and communication patterns between single cells by quantifying cell–cell spatial relationships. Using permutation testing, we assigned the likelihood of interaction or avoidance behaviours between cell pairs across LUAD architectures ranging from least to most aggressive (Fig. 2c,d). Tumour cells had a universal tendency towards homotypic interactions and a relative avoidance with other cell types (Fig. 2d), consistent with spatial analyses in breast cancer^12^. Homotypic interactions were high across several immune cell populations, suggestive of a spatially coordinated TIME. Many of these interactions were discordant with the pattern of cell frequencies, revealing that the spatial relationship of cell–cell interactions may hold greater prognostic value than frequency alone. In higher-grade histological patterns (solid and micropapillary), both neutrophils and endothelial cells had an increased tendency for interactions with cancer cells compared with lower-grade subtypes (Fig. 2d, box 1a,b). These relationships are consistent with the ability of neutrophils to facilitate tumour cell extravasation into blood vessels, thereby promoting haematogenic metastasis^26,27^, and solid-predominant LUAD has the highest rate of metastasis compared with other histologies^28^.

In low-grade lepidic and papillary tumours, CD8^+^ and CD4^+^ T cells had a stronger tendency for interaction with cancer cells than high-grade solid LUAD (Fig. 2c,d, box 2), despite the fact that overall CD8^+^ and CD4^+^ T cell frequencies were not associated with progression (Fig. 2b, box 6). This relationship echoes previous findings that the spatial interaction of T cells and tumour cells is a stronger indicator of non-recurrence than T cell density alone^29^. Moreover, despite the co-occurrence of CD163^+^ macrophages and CD8^+^ T cells in low-grade tumours (Extended Data Fig. 5c, box 2), their tendency to directly interact was strongest in high-grade tumours and decreased as tumors became lower grade (Fig. 2d, box 3). This is consistent with the role for CD163^+^ macrophages in suppressing CD8^+^ T cell function within the TIME^30^. Similarly, B cells exhibited a greater tendency to interact with CD163^+^ macrophages in high-grade tumours (Fig. 2d, box 4), despite the observation that high B cell frequency was indicative of prolonged survival (Fig. 2b, box 3). In patient tumours specifically enriched in mature antigen-presenting CD40^+^ B cells^31^, these cells became generally more interactive across TIME populations (Extended Data Fig. 5e). Finally, endothelial cells tended to interact with many immune populations in high-grade tumours compared with low-grade tumours, including CD163^+^ macrophages and monocytes (Fig. 2d, box 5); these interactions may be reminiscent of innate regulation of vascular inflammation, consistent with our observation that immune infiltration was highest in the solid pattern (Fig. 1g), driven largely by differences within the myeloid compartment (Fig. 1h). Together, these analyses paint an overall picture of how stromal interactions shift among histological patterns and exemplify how spatial relationships, rather than cell frequency alone, are important to understand TIME biology.

## LUAD architecture and survival outcomes

**Fig. 3:**
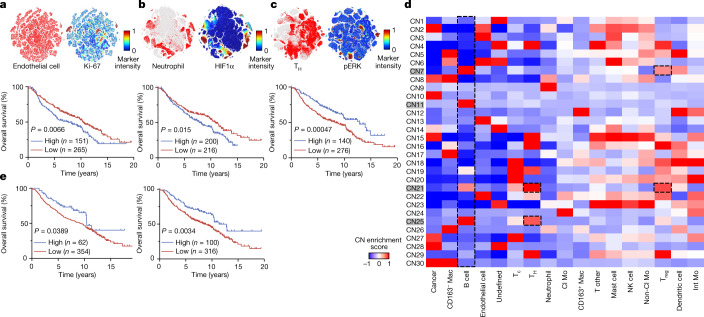
Single-cell populations and neighbourhoods are associated with distinct outcomes in LUAD. **a**–**c**, *t*-SNE of endothelial, myeloid and lymphoid cell populations highlighting the distribution of 108,387 endothelial cells (**a**), 42,427 neutrophils (**b**) and 147,980 CD4^+^ T_H_ cells (**c**), and the positivity of the Ki-67 (endothelial cells), HIF1α (neutrophils) and pERK (T_H_ cells) markers. Kaplan–Meier curves of overall survival for 416 patients with LUAD based on low (*z-*score < 0) and high (*z*-score ≥ 0) prevalence of the indicated cell types are also shown. **d**, Heatmap of 30 CNs discovered in 416 patients with LUAD. The CNs highlighted in grey refer to B-cell-enriched neighbourhoods. **e**, Kaplan–Meier curves of overall survival for 416 patients with LUAD based on low (*z*-score < 0) and high (*z*-score ≥ 0) prevalence of B cell CN11 (left) and CN25 (right). log-rank test was used for statistical analysis (**a**–**c**,**e**).

**Extended Data Fig. 6:**
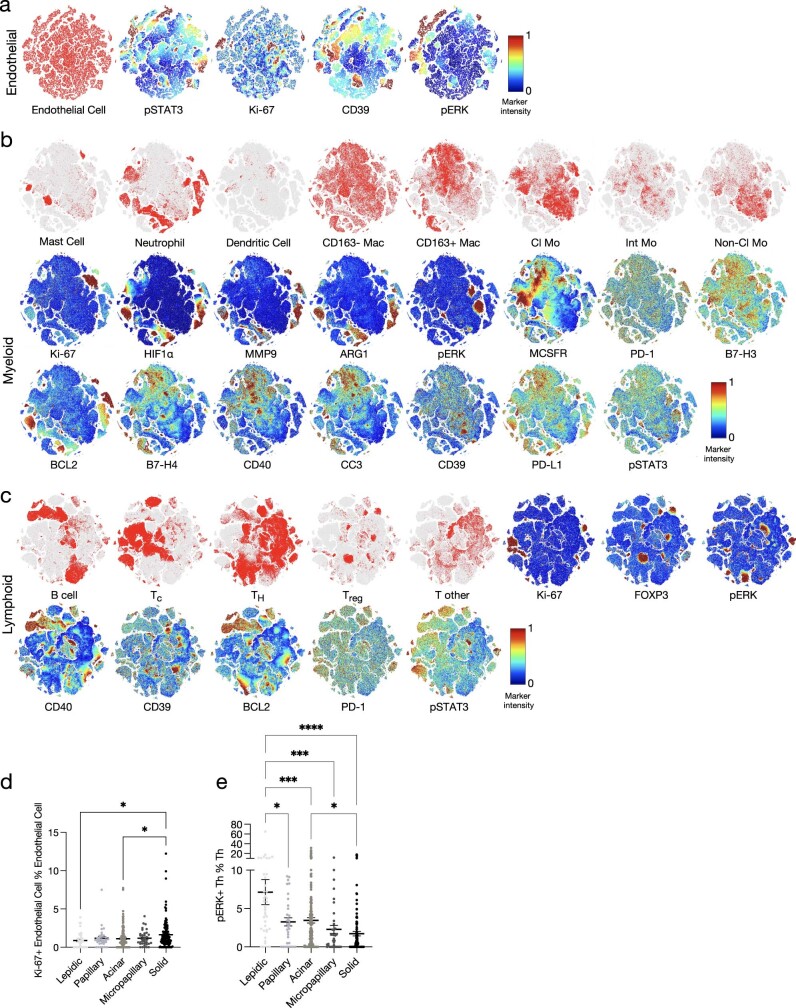
Activation markers and single-cell phenotypes in lung adenocarcinoma. **a**, T-distributed stochastic neighbour embedding (t-SNE) of 108,387 endothelial cells. pSTAT3, Ki-67, CD39 and pERK expression within the endothelial cluster is shown. **b**, t-SNE plots of 9,480 mast cells, 42,427 neutrophils, 1,407 dendritic cells, 10,000 subsampled CD163- macrophages, 39,502 CD163^+^ macrophages, 37,653 classical monocytes, 8,330 intermediate monocytes and 17,029 non-classical monocytes. Ki-67, HIF1α, MMP9, ARG1, pERK, MCSFR, PD-1, B7-H3, BCL2, B7-H4, CD40, CC3, CD39, PD-L1 and pSTAT3 expression in the myeloid compartment is shown. **c**, t-SNE plots of 62,941 B cells, 98,396 T_c_, 147,980 T_H_, 19,839 T_reg_ and 23,995 T other cells. Ki-67, FOXP3, pERK, CD40, CD39, BCL2, PD-1 and pSTAT3 expression in the lymphoid compartment is shown. **d-e**, Prevalence of Ki-67^+^ endothelial cells (Comparison between Lepidic and Solid: * P = 0.0362. Comparison between Acinar and Solid: * P = 0.0185) and pERK+ T_H_ cells (Comparison between Lepidic and Solid: **** P = <0.0001. Comparison between Lepidic and Micropapillary: *** P = 0.0004. Comparison between Lepidic and Acinar: *** P = 0.0003. Comparison between Lepidic and Papillary: * P = 0.0102. Comparison between Acinar and Solid: * P = 0.0288) as a proportion of endothelial and T_H_ cells respectively, across histological subgroups in 416 lung adenocarcinoma patients (Lepidic n = 40, Papillary n = 33, Acinar n = 190, Micropapillary n = 35, Solid n = 118). Mean ± SEM. Statistical analysis (**d-e:** one-way ANOVA with Tukey multiple comparison test).

To complement our analyses of cell frequencies and interactions, we next explored how cellular phenotypes within the microenvironment relate to survival. We extracted all microenvironmental populations represented in our dataset (including endothelial, myeloid or lymphoid compartments) and performed *t*-distributed stochastic neighbour embedding (*t*-SNE) based on functional markers in our antibody panel (Extended Data Fig. 6a–c). First, outside the immune compartment, we observed a distinct population of proliferative Ki-67^+^ endothelial cells, whose frequency was associated with poor overall survival (Fig. 3a and Supplementary Table 5) and high-grade solid tumours (Extended Data Fig. 6d). Proliferation of the endothelium underlies angiogenesis in response to hypoxia, a common feature of aggressive tumours^32^. We therefore explored vascular interactions in high-grade patterns and found an enrichment in endothelial cell interactions with neutrophils (Fig. 2d, box 6), leading us to question how specific neutrophil subsets may respond to hypoxic conditions. We observed several neutrophil states based on the expression pattern of three markers: HIF1α^+^, ARG1^+^ and MMP9^+^ (Fig. 3b and Extended Data Fig. 6b). Despite a high frequency of total neutrophils not being correlated with survival in our cohort (Fig. 2b, box 7), an increase in the proportion of the HIF1α^+^ subset was significantly associated with worse overall survival (Fig. 3b and Supplementary Table 5), which may reflect cases in which angiogenesis is insufficient to alleviate hypoxia. Neutrophils and other granulocytes are sensitive to low-oxygen conditions, and can adopt immunosuppressive behaviours against T cells in this setting^33^. Indeed, we observed that neutrophils exhibit a higher tendency to interact with immunosuppressive T_reg_ cells in high-grade tumours (Fig. 2d, box 7). Phenotypic analysis within the lymphoid compartment revealed active ERK signalling within a subset of CD4^+^ T cells associated with prolonged survival (Fig. 3c and Supplementary Table 5), which is known to suppress differentiation into T_reg_ cells^34^. Consistently, pERK^+^CD4^+^ T cells were enriched in low-grade lepidic tumours (Extended Data Fig. 6e) where neutrophil–T_reg_ cell interactions were the lowest (Fig. 2d, box 7), and reduced in high-grade solid tumours (Extended Data Fig. 6e) where T_reg_ cells were most abundant (Fig. 2b, box 8). Together, these findings provide a snapshot of spatially resolved phenotypic programs associated with more aggressive tumours, as they relate to tumour hypoxia and an immunosuppressive niche.

**Extended Data Fig. 7:**
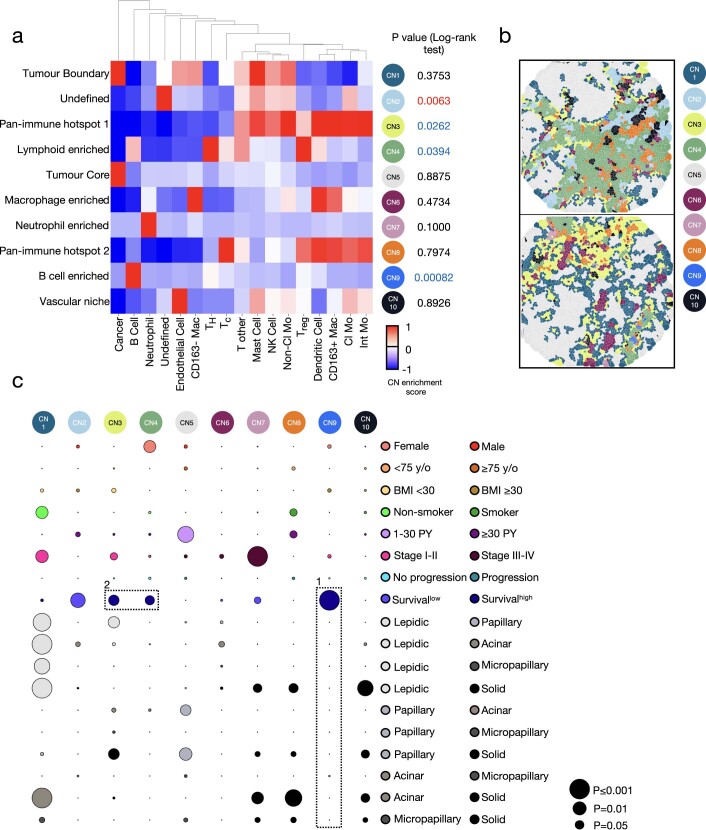
Variability in 10 cellular neighbourhoods across clinical variables in lung adenocarcinoma. **a**, Heatmap of 10 cellular neighbourhoods discovered in 416 lung adenocarcinoma patients. **b**, Representative images of 10 cellular neighbourhoods using Voronoi diagrams. **c**, Bubble plot where circle colour indicates which of the two comparisons on the y-axis has higher levels of the cell type on the x-axis (Female n = 233, Male n = 183), age (<75 yo n = 369, ≥75 yo n = 47), BMI (<30 n = 346, ≥30 n = 70), smoking status (Smoker n = 376, Non-smoker n = 38), pack-years (1—30 n = 89, ≥30 n = 256), stage (I-II n = 365, III-IV n = 50), progression status (Progression n = 64, No progression n = 340) and histological subgroup (Lepidic n = 40, Papillary n = 33, Acinar n = 190, Micropapillary n = 35, Solid n = 118). The size of the circle represents the level of significance. Survival^low^ refers to survival in the context of low (z score <0) prevalence of depicted 10 cellular neighbourhoods. The black boxes depict associations referenced in the text. For exact P values, see Supplementary Table 6. Statistical analysis (**a:** log-rank test, **c:** FDR-corrected two-tailed Student t-test for sex, age, BMI, smoking status, pack-years, stage, progression status; one-way ANOVA with Tukey multiple comparison test for histological subgroup; log-rank test for survival).

Beyond pairwise interactions, our data hint at the existence of larger cellular communities that are distinctively organized within the TIME across LUAD subtypes. To assess this, we followed a canonical approach to establish cellular neighbourhoods by first identifying the ten nearest spatial neighbours for each individual cell^12,14^. We then reclassified cells on the basis of their spatially defined cellular neighbourhood (CN). Using this approach, we discovered ten CNs that recapitulated both new and known tissue architectures, which we named: tumour boundary (CN1), undefined (CN2), pan-immune hotspot 1 (CN3), lymphoid enriched (CN4), tumour core (CN5), macrophage enriched (CN6), neutrophil enriched (CN7), pan-immune hotspot 2 (CN8), B cell enriched (CN9) and vascular niche (CN10) (Extended Data Fig. 7a,b). To identify CNs associated with survival, we performed Kaplan–Meier analysis by designating the frequency of CNs for each patient as high (CN^high^; *z*-score ≥ 0) or low (CN^low^; *z*-score < 0). Consistent with our findings related to B cell frequency (Fig. 2b, box 3, and Supplementary Table 4), CN9^high^ (B cell-enriched) was significantly associated with increased overall survival (Extended Data Fig. 7a and Supplementary Table 6), despite minimal differences in CN9 representation across histological patterns (Extended Data Fig. 7c, box 1). CN3^high^ (pan-immune hotspot 1) and CN4^high^ (lymphoid enriched) were also significantly correlated with increased overall survival across LUAD histologies (Extended Data Fig. 7a,c, box 2). When survival was analysed within histological patterns, associations with increased survival were noted for CN4^low^ (lymphoid enriched) within the lepidic pattern, CN9^high^ (B cell enriched) in the acinar pattern and CN2^low^ (undefined) and CN4^high^ (lymphoid enriched) in the solid pattern (Supplementary Fig. 2).

**Extended Data Fig. 8:**
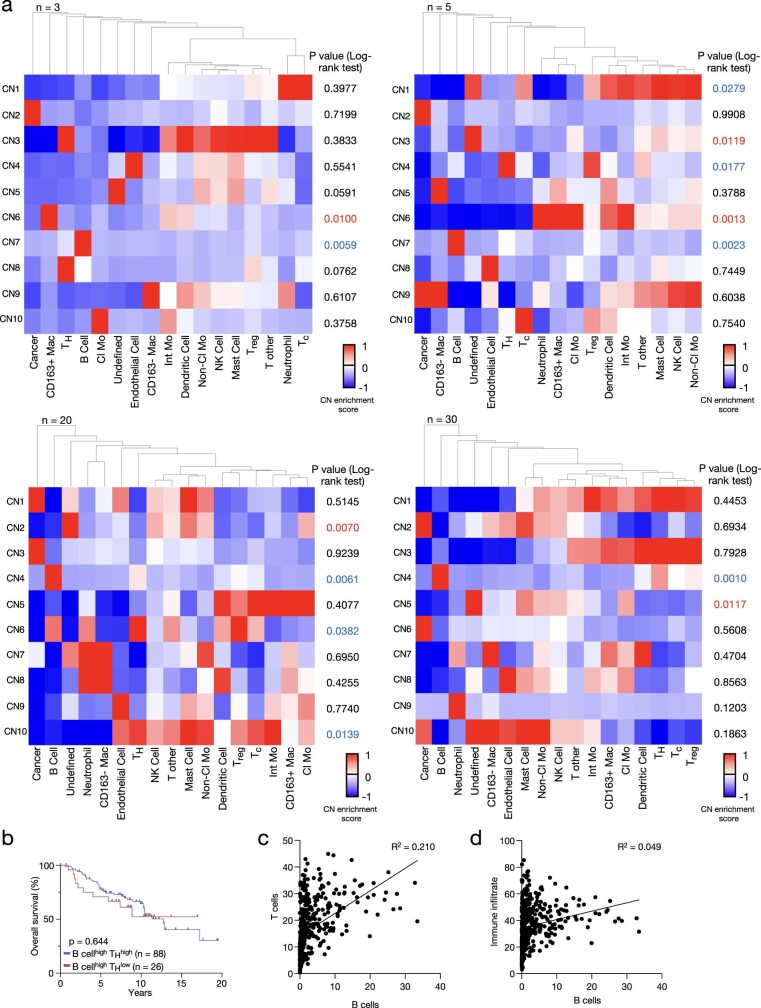
Heatmaps of 10 cellular neighbourhoods with 3, 5, 20 and 30 closest cells (n) discovered in 416 lung adenocarcinoma patients. **a**, Tables depict result of log-rank test of the overall survival for 416 lung adenocarcinoma patients based on low (z score <0) and high (z score ≥0) prevalence. Black p values indicate no significant difference (p ≥ 0.05) in survival between the two groups. Blue p values indicate better survival (p < 0.05) with high prevalence and red p values indicate better survival (p < 0.05) with low prevalence of the depicted groups. **b**, Kaplan–Meier curves of overall survival for lung adenocarcinoma patients based on low (z score <0) and high (z score ≥0) prevalence of T_H_ cells and high (z score ≥0) prevalence of B cells. **c**, Correlation of T cell and B cell prevalence. **d**, Correlation of immune infiltrate and B cell prevalence. Statistical analysis (**b:** Log-rank test).

**Extended Data Fig. 9:**
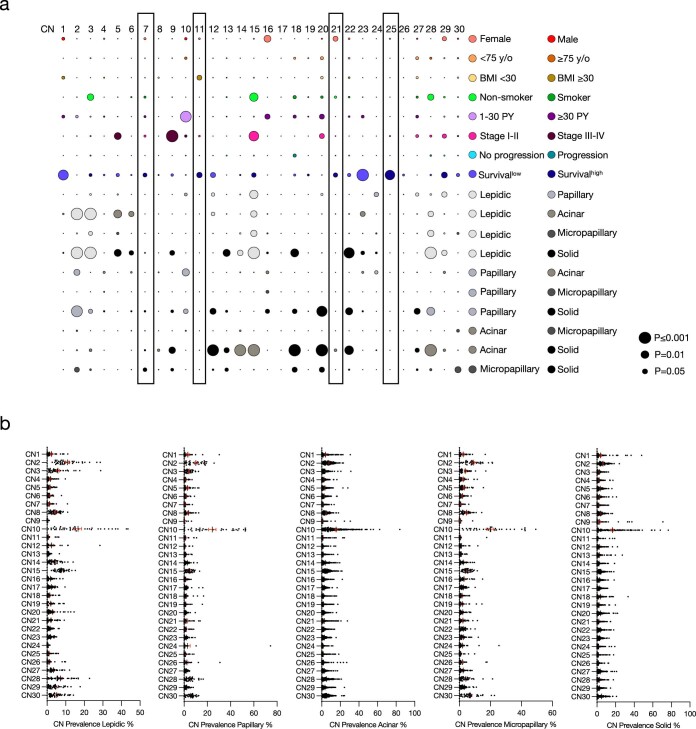
Variability in 30 cellular neighbourhoods across clinical variables in lung adenocarcinoma. **a**, Bubble plot where circle colour indicates which of the two comparisons on the y-axis has higher levels of the cell type on the x-axis (Female n = 233, Male n = 183), age (<75 yo n = 369, ≥75 yo n = 47), BMI (<30 n = 346, ≥30 n = 70), smoking status (Smoker n = 376, Non-smoker n = 38), pack-years (1-30 n = 89, ≥30 n = 256), stage (I-II n = 365, III-IV n = 50), progression status (Progression n = 64, No progression n = 340) and histological subgroup (Lepidic n = 40, Papillary n = 33, Acinar n = 190, Micropapillary n = 35, Solid n = 118). The size of the circle represents the level of significance. Survival^low^ refers to survival in the context of low (z score <0) prevalence of depicted 30 cellular neighbourhoods. For exact P values, see Supplementary Table 7. The black boxes depict associations referenced in the text. **b**, Prevalence of 30 cellular neighbourhoods across 5 histological subtypes (Lepidic n = 40, Papillary n = 33, Acinar n = 190, Micropapillary n = 35, Solid n = 118). Mean ± SEM. Statistical analysis (**a:** FDR-corrected two-tailed Student t-test for sex, age, BMI, smoking status, pack-years, stage, progression status; one-way ANOVA with Tukey multiple comparison test for histological subgroup; log-rank test for survival).

We were particularly interested in dissecting B cell neighbourhoods in greater detail, given the prognostic value of B cells in our dataset (Fig. 2b, box 3). Two variables that affect CN analysis include the number of interacting cells within a neighbourhood (denoted as *n*) and the number of total neighbourhoods (denoted as tCN). To further explore the spatial relationship between CNs and survival, we first altered the number of nearest spatial neighbours for each individual cell (*n*) while maintaining a constant number of neighbourhoods (tCN = 10). Across a wide range of *n* values (*n* = 3–30), CNs enriched in B cells were significantly associated with survival (Extended Data Fig. 8a), with the most significant association resulting from *n* = 10 and tCN = 10 (Extended Data Fig. 7a). To resolve B cell interactions that drive this survival advantage, we increased the tCN to 30. Using this approach, we were able to resolve four B cell-enriched neighbourhoods (CN7, CN11, CN21 and CN25) (Fig. 3d). Across these neighbourhoods, the survival advantage associated with B cells was negated when CNs concurrently displayed an enrichment in T_reg_ cells (CN7 and CN21), whereas the survival advantage was maintained for CN11 (*P* = 0.0389) and CN25 (*P* = 0.0034) where T_reg_ cells were lower (Fig. 3e and Supplementary Table 7). When comparing these two neighbourhoods, we noted a greater survival advantage for CN25, which was also enriched for CD4^+^ helper T cells (by contrast, CN11 was enriched for B cells alone). To determine whether the improved survival benefit associated with CN25 was related to the interaction of B cells and CD4^+^ helper T cells or to the prevalence of both cell types independent of their interaction, we plotted the survival association of patients who were B cell-high and CD4^+^ helper T cell-high versus patients who were B cell-high and CD4^+^ helper T cell-low and observed no significant difference (*P* = 0.644; Extended Data Fig. 8b). Moreover, the correlation between T cells and B cells in our cohort was low with an *R*^2^ of 0.210, thus making it less likely that CD4^+^ helper T cells and B cells are interacting as a result of a strong correlation in the prevalence of both cell types (Extended Data Fig. 8c,d). These data suggest that the improved survival association for CN25 is related to the interaction of B cells and CD4^+^ helper T cells beyond the prevalence of both cell types alone. However, an enrichment in T_reg_ cells was still sufficient to negate this survival benefit (CN21), emphasizing the importance of multicellular B cell interactions within the TIME. Finally, there was no significant association between these B cell-enriched CNs and any other clinical variable, including histological subtype (Extended Data Fig. 9a,b and Supplementary Table 7). Together, these findings suggest that the spatial organization of TIME interactions may provide additional insight into individual patient survival beyond histological subtype classifications and cell prevalence.

## Predicting outcomes using deep learning

**Fig. 4:**
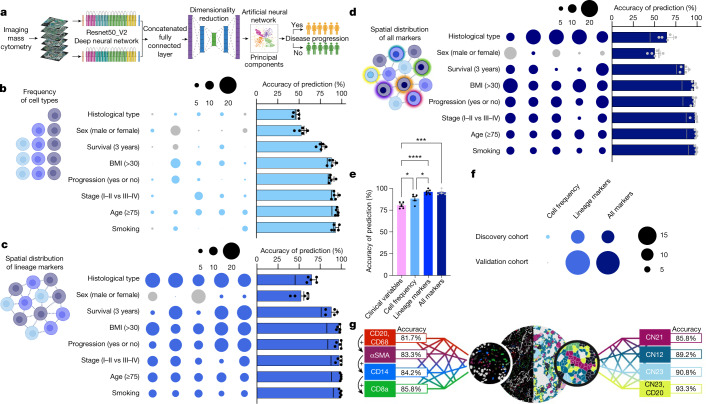
Machine learning of IMC data predicts clinical outcomes. **a**, Schematic of the deep-learning-based strategy involving deep residual networks (Resnet50) architecture on the ImageNet dataset for feature extraction from IMC image channels. **b**–**d**, Fivefold cross-validation across clinical outcomes: histological patterns, sex (male or female), BMI (less than 30 or 30 or higher) and age (younger than 75 years of age or 75 years of age or older) *n* = 416; survival (less than 3 years, 3 years or longer) *n* = 407; progression status (progression or no progression) *n* = 404; stage (I–II or III–IV) *n* = 415; smoking status (smoker or non-smoker) *n* = 414 using frequency of cell types (**b**); spatial distribution of lineage markers (**c**) and spatial distribution of all markers (**d**). The size of the bubble represents deviation from baseline, with blue and grey indicating an improvement or worsening in predictive performance, respectively. The line in the bar plot represents the baseline. Schematics in **a**–**d** were created with BioRender. **e**, Accuracy of clinical progression prediction in patients with stage I LUAD (*n* = 286) using clinical variables, cell frequency, lineage marker and ‘all markers’ models. Comparison between the clinical variables and the cell frequency model: *P = 0.0319. Comparison between the clinical variables and lineage marker model: ****P < 0.0001. Comparison between the clinical variables and all markers model: ***P = 0.0001. Comparison between the cell frequency and lineage marker model: *P = 0.0321. **f**, Accuracy of clinical progression prediction in patients with stage I LUAD; discovery cohort (*n* = 286) and validation cohort (*n* = 60; 120 cores) in the cell frequency, lineage and all markers models compared with baseline. The size of the bubble represents deviation from baseline, with blue and grey indicating an improvement or worsening in predictive performance, respectively. **g**, Accuracy of clinical progression prediction in patients with stage I LUAD (validation cohort *n* = 60; 120 cores) using combinations of top-ranked (left) and neighbourhood-derived (right) lineage markers. For all combinations, see Supplementary Table 15. Data shown as mean ± s.e.m. (**b**–**e**). One-way ANOVA with Tukey multiple comparison test was used for statistical analysis (**e**).

Given our finding that spatial neighbourhoods are predictive of survival regardless of LUAD architectures, we wondered whether we could leverage spatial data to predict clinical outcomes by using a deep-learning approach (Fig. 4a). We took advantage of transfer learning by using a pretrained convolutional neural network model. We chose the deep residual networks^35^ architecture pretrained on the ImageNet dataset^36^. Using the *k*-fold cross-validation method, we split the data into five folds, with 20% of the data for each fold. In our experiments, we considered four of the folds (80% of the patients) as the training data and the remaining fold (20%) for testing to evaluate the prediction accuracy. We repeated this for all possible combinations. For proof of principle, we first assessed whether the frequency of cells alone within each image would be sufficient to predict clinical variables. We tested routine clinical variables that demonstrated some variation in cell-type frequencies including histological subtype, sex, survival, BMI, cancer progression, cancer stage, age and smoking. Our goal was to increase the ability to predict clinical outcomes above the baseline prediction score, which reflects the chance of predicting the major class over the total number of examples involved for that specific variable. However, we saw negligible increases in prediction score above baseline for most of the clinical variables, suggesting that cell frequency alone does not capture the tumour architecture with enough resolution to predict clinical variables with high confidence (Fig. 4b and Supplementary Table 8).

We next investigated whether the integration of spatial information, obtained by inputting all the raw lineage marker images into our model, would help recognition performance. Using this approach, we observed a significant boost in performance for all clinical variables tested compared with cell frequency alone, suggesting that single-cell positional information that is encoded in each of the multiplex image scans is critical to interpreting the complex TIME that underlies clinical correlates (Fig. 4c and Supplementary Table 9). Spatial information did not confer an increase above baseline for sex, indicating that the TIME that underlies tumours from male individuals versus female individuals is indistinguishable using our model. Finally, to compare whether additional channels from our IMC images would lead to better recognition performance (that is, integration of spatially resolved immune functional substates), we used all the markers in our panel and repeated the predictions. The additional channels did not boost performance, suggesting that there is a certain threshold beyond which additional markers do not add value to clinical predictions based on our model (Fig. 4d and Supplementary Table 10).

**Extended Data Fig. 10:**
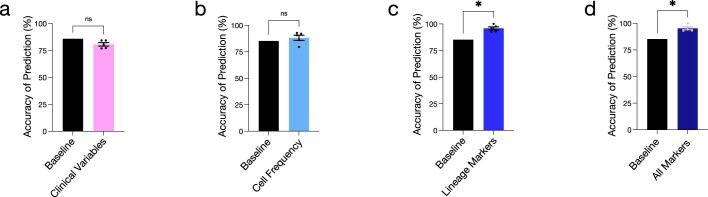
Machine learning of imaging mass cytometry data improves prediction of progression. Accuracy of clinical progression prediction in stage I lung adenocarcinoma patients (n = 286) in the **a**, clinical variables; **b**, cell frequency; **c**, lineage markers and **d**, “all markers” models. Comparison between baseline and the lineage marker model: * P = 0.0343. Comparison between baseline and the “all marker” model : * P = 0.0355. Mean ± SEM. Statistical analysis (**a—d:** two-tailed Student t-test).

We next sought to leverage our deep-learning approach to address a clinically meaningful problem. Although adjuvant chemotherapy has long been demonstrated to improve overall survival in patients with NSCLC who have stage IIA to IIIA disease, patients with stage I tumours smaller than 4 cm currently do not receive adjuvant chemotherapy^37,38^, despite that many of these patients are at increased risk for progression^39^. In fact, these patients currently have minimal approved peri-adjuvant therapeutic options^37^. Even with complete lung tumour resection, a significant proportion of these patients will relapse^39^. Therefore, we next assessed whether we could predict progression in patients with stage IA–IB lung cancer. The use of standard clinical information in our model (sex, age, BMI, smoking status, pack-years, surgery type, maximum tumour size, tumour grade, predominant histological pattern and stage) was insufficient to predict progression over baseline (Fig. 4e, Extended Data Fig. 10a and Supplementary Table 11). Consistent with our previous results, the frequency of cells alone was also insufficient to significantly predict progression (Fig. 4e, Extended Data Fig. 10b and Supplementary Table 11). When we included spatial information, however, our model predicted progression with 95.9% accuracy from a single 1-mm^2^ tumour core, smaller than most standard needle biopsies used to establish a diagnosis of lung cancer (Fig. 4e, Extended Data Fig. 10c and Supplementary Table 11). Additional markers, again, did not boost the prediction accuracy (Fig. 4e, Extended Data Fig. 10d and Supplementary Table 11), indicating that there is a minimum threshold of markers required to ascertain accurate predictions, a promising finding for translational practicality.

To validate our findings and to assess how heterogeneity within the lung TIME may affect our predictions, we performed IMC on an independent validation cohort consisting of 60 patients with primary LUAD that included two spatially distinct cores per tumour (Supplementary Table 12). In this new dataset, after training on our discovery cohort, the use of raw images from our lineage markers was able to predict progression with high accuracy (94.2% accuracy; Fig. 4f and Supplementary Table 13), with no added benefit from integrating the entire panel (93.3% accuracy). Of note, our validation cohort was more balanced, with a lower baseline prediction score (75.0%), confirming that there are spatially defined features within these images that probably reflect clinical outcomes. When assessing intratumour heterogeneity, we found substantial agreement in the predictions between two distinct cores from the same tumour (91.7%, lineage markers model). Despite these promising results, we acknowledge that tumour heterogeneity remains a challenge for accurate clinical and pathological diagnosis, an area of research that may benefit greatly from the application of artificial intelligence approaches.

One limitation of highly multiplexed imaging is the impracticality of translating discoveries into a clinically actionable assay that is broadly accessible. We thus sought to determine the minimum threshold of markers that could be used to predict progression without compromising prediction accuracy, with the goal of reducing our panel to approximately five markers (which is more likely to be amenable to clinical pathology practice). We first assessed the predictive performance of the spatial information derived from each individual marker in our antibody panel. Not surprisingly, we found that CD20 (a B cell marker) was most associated with an improved prediction of progression in our discovery cohort (Supplementary Table 14). Next, on the basis of the ranking of individual prediction scores, we combined the top two, three, four or five markers and tested whether combinations could predict progression in our validation dataset with high accuracy. Using this approach, we did not reach the level of accuracy that was achieved when all lineage markers were used (Fig. 4g and Supplementary Table 15). As an alternative approach, we took advantage of the spatial information embedded within our dataset, by using our CN analysis as a guide to identify rational combinations of markers whose spatial distribution are strongly correlated with survival (Fig. 3d and Supplementary Table 7). We reasoned that specific interactions may have prognostic value and would therefore be informative in predicting progression. Using this approach, we discovered that the combination of five markers—CD14, CD16, CD94, αSMA and CD117 (enriched in CN23, the neighbourhood most significantly associated with overall survival)—resulted in 90.8% accuracy (Fig. 4g and Supplementary Table 15). When we added CD20 (the individual marker demonstrating the highest predictive potential for progression), we increased accuracy to 93.3%, with 95.6% precision and recall. Overall, these data suggest that spatially resolved single-cell datasets may be highly valuable in the future to help to inform personalized peri-operative care plans to minimize toxicity for those destined to be cured, or to increase cure rates for those destined to recur.

## Discussion

Here we applied highly multiplexed IMC to characterize the cellular landscape of the LUAD TIME. We identified cellular dynamics and spatial features that correlate with distinct clinical outcomes including patient survival. Our data represent a valuable resource that adds to a quickly evolving body of literature supporting the importance of spatially resolved single-cell datasets in understanding how the TIME architecture relates to tumour biology. As lung cancer remains by far the largest cause of cancer-related death, there is untapped value in combining single-cell technology with deep-learning approaches to develop intelligent predictive algorithms to help to triage patients onto the therapeutic regimens that are best suited for their individual cancer. Our findings utilize a 5-µm section of a single 1-mm^2^ core of formalin-fixed paraffin-embedded tumour tissue to predict recurrence with high accuracy, which can be obtained from surgical resection or a biopsy. Nevertheless, clinical sampling bias remains a challenge in studies in which small regions of tumours are captured within a small amount of material. Future work will focus on using lower-plex technologies while attempting to maintain predictive accuracy to achieve translational feasibility. Our findings represent an important advance over existing prediction tools that use clinical and pathological variables and may enable more effective utilization of a growing armamentarium of peri-adjuvant systemic therapies to improve cancer outcomes^40,41^.

## Methods

### Clinical cohort

A cohort of 416 patients with LUAD were included in this study with follow-up time ranging from February 1996 to July 2020. For the validation cohort, 60 patients with LUAD with follow-up time ranging from February 2012 to May 2022 were included with two distinct cores per patient. All samples obtained were primary treatment-naive LUADs diagnosed by a board-certified pathologist following surgical resection or biopsy. Clinical information on all patients included can be found in Supplementary Tables 1 and 12. Tissue microarrays were constructed by selecting one 1-mm^2^ core from the surgical tumour specimen. Patient samples and clinical information were obtained following written informed patient consent. The protocols for human sample biobanking were approved (ethics, scientific and final) through the IUCPQ Biobank, protocol number IRB #2022-3474, 22090, and the MUHC protocol numbers IRB #2014-1119 and 2019-5253.

### Sample staining and IMC

Formalin-fixed paraffin-embedded (FFPE) slides were deparaffinized at 70 °C by incubation in EZ Prep solution (Roche Diagnostics) followed by antigen retrieval at 95 °C in standard cell conditioning 1 solution (Roche Diagnostics). The Ventana Discovery Ultra auto-stainer platform (Roche Diagnostics) was used for antigen retrieval. Slides were rinsed with 1× PBS and incubated for 45 min in Dako serum-free protein block solution (Agilent). Slides were stained with a cocktail containing metal-tagged antibodies at optimized dilutions overnight at 4 °C. All conjugations were performed by the Single Cell and Imaging Mass Cytometry Platform at the Goodman Cancer Institute (McGill University), using Maxpar Conjugation Kits (Fluidigm). Information on the antibodies used can be found in Supplementary Table 2. Slides were then washed with 0.2% Triton X and 1× PBS. An optimized dilution of the secondary antibody cocktail containing metal-conjugated anti-biotin was prepared in Dako antibody diluent. After a 1-h incubation, slides were washed with 0.2% Triton X and 1× PBS. Before IMC acquisition, Cell-ID Intercalator-Ir (Fluidigm) at a dilution of 1:400 was used to counterstain slides in 1× PBS for 30 min at room temperature. Slides were then rinsed for 5 min with distilled water and air-dried. IMC images were acquired at a resolution of roughly 1 μm. Cores were laser-ablated at a frequency of 200 Hz using the Hyperion Imaging System (Fluidigm) and raw data were compiled using the Fluidigm commercial acquisition software. Of note, in our validation cohort, we stained with alpha cleaved H3 (176Yb) instead of histone H3 (176Yb). Accordingly, this marker was excluded from validating our deep-learning predictions of progression.

### Antibody optimization

Antibodies were optimized on control tissues including the spleen, tonsil, appendix, placenta, thymus, normal lung and LUAD. Multiplex quality-control staining of positive and negative control tissue can be seen in Extended Data Figs. 2–4, with four representative images staining for each of the 35 markers in our panel.

### Data transformation and normalization

Data presented were not transformed. All analyses were based on raw IMC measurements. For heatmap visualization, expression data were normalized to the 95th percentile and *z*-scored cluster means were plotted. Single-cell marker expressions were summarized by mean pixel values for each channel.

### Cell segmentation and lineage assignment

All markers underwent a staining quality check before cell segmentation (Extended Data Figs. 2–4). A small number of markers did not consistently stain every sample in our cohort, so we chose not to make any conclusions based on those markers (GM-CSFR, PD-1, PD-L1 and B7-H3). Note that CD163 (a putative ‘M2-like’ marker) was chosen to subdivide macrophage populations on the basis that this marker is often upregulated in tumour-associated macrophages and has been used to categorize macrophages in multiplex imaging studies^14,42,43^. Although the terms ‘M1/pro-inflammatory’ and ‘M2/anti-inflammatory’ have traditionally been used to classify macrophage activation states, these terms are outdated and were therefore avoided^44,45^. Using a novel cell segmentation pipeline that combines classical and modern machine-learning-based computer vision algorithms, we segmented all cells contained within the IMC images. The model used is a fully automated hybrid cell detection model that eliminates subjective bias and enables high-throughput image segmentation. It allows us to accurately distinguish cells across diverse tissue microenvironments and resolve low-resolution structures. The details of our image segmentation approach can be found here: https://biorxiv.org/cgi/content/short/2022.02.27.482183v1. Owing to existing phenotyping challenges for highly multiplexed imaging, we created a cell phenotyping pipeline to assign cell phenotypes. Our strategy relies on canonical lineage markers and uses a supervised hierarchal approach that integrates the staining quality, the expected population abundance and cell lineage maturation to assign cells. We used *k*-means clustering^46^ and a mixture of generalized Gaussian models^47^ to generate a mask or level for each marker within a multi-level image stack created based on staining intensity. This allowed us to evaluate the existence of a marker at a particular location. Each marker in our panel was assessed using six levels and the appropriate mask was subsequently manually curated for each marker. Each mask is produced using the following procedure:

1. The greyscale image channel is convolved with a median filter with a particular window size (3 × 3).
2. Each pixel in the image is clustered into six groups of intensity levels using the *k*-means algorithm.
3. For each channel, we then selected all groups up to a particular level as foreground (1) and the rest as background (0).
4. To obtain smoother binary masks, we also applied a morphological blob removal process in which binary blobs of a particular area are removed from masks to avoid noisy regions.
5. To further refine the accuracy of select markers, additional channel-specific morphological operations were applied by computing an additional binary mask obtained using the adaptive binarization method with a sensitivity of 0.4. This mask is then amalgamated with the mask obtained in step 4.

As a formula, for each cell ${c}_{i}$, we consider the curated mask for each lineage marker ${M}_{k}$, where $k=1,\ldots ,n$ and *n* is the number of lineage markers. Let us assume ${p}_{{c}_{i}}^{j}$ be the *j*th pixel that lies in the surrounding of ${c}_{i}$ and each pixel has the following presence vector based on the lineage markers:

$$E({p}_{{c}_{i}}^{j})=\{{p}_{{M}_{1}}^{j},{p}_{{M}_{2}}^{j},\ldots ,{p}_{{M}_{n}}^{j}\}$$

where ${p}_{{M}_{i}}=\{0\,\text{or}\,1\}$, which determines whether the pixel ${p}_{{c}_{i}}^{j}$ is positive for a particular marker. Next, to determine whether each pixel within a cell is positive or negative for a given marker, we determined the majority vector by summing the presence of all vectors as:

$${M}_{{c}_{i}}=\left\{\mathop{\sum }\limits_{j=1}^{{N}_{{c}_{i}}}{p}_{{M}_{1}}^{j},\mathop{\sum }\limits_{j=1}^{{N}_{{c}_{i}}}{p}_{{M}_{2}}^{j},\ldots ,\mathop{\sum }\limits_{j=1}^{{N}_{{c}_{i}}}{p}_{{M}_{n}}^{j}\right\}$$

where ${N}_{{c}_{i}}$ is the number of pixels in the cell ${c}_{i}$. The maximum value in vector ${M}_{{c}_{i}}$ determines the cell-type assignment. Cell lineages were assigned in rank priority order (Extended Data Fig. 1c). See the ‘Code availability’ section for additional details.

### Cell–cell pairwise interaction

We performed a permutation-test-based analysis of spatial single-cell interactions to identify significant pairwise interaction–avoidance between cells^12^. Interacting cells were defined as those within six pixels. *P* values less than 0.01 were deemed significant.

### Neighbourhood identification

To generate CNs, we used a ‘window’ capture strategy consisting of the number of cells (*n*) in closest proximity to a given cell as previously described^14^. Each window is a frequency vector consisting of the types of X (as indicated) closest cells to a given cell. Obtaining all the window vectors for each cell, initial cells (Extended Data Fig. 7a) were clustered using Scikit-learn, a software machine-learning library for Python, and MiniBatchKMeans clustering algorithm version 0.24.2 with default batch size = 100 and random_state = 0. Subsequent CN analysis was performed using the MiniBatchKMeans clustering algorithm version 1.1.2 with default batch size = 1,024 and random_state = 0. Every cell was subsequently allocated to a CN based on their defining window. The prevalence of each neighbourhood in each core was normalized so that the sum of neighbourhood prevalence for that core was 100%. Values were then *z*-scored and cores with a *z*-score above or equal to 0 and below 0 were compared for survival outcomes.

### *t*-SNE

All *t*-SNE plots were generated in MATLAB (version 2019b) using default parameters. For visualization, expression data were normalized to the 95th percentile.

### Deep learning

All deep-learning analysis steps were performed in Python (version 3.7.12). We used the TensorFlow (version 2.8.0) framework alongside Keras, which now acts as an interface for the TensorFlow library. We have two modes of data for our experiments: (1) raw IMC images, and (2) cell frequencies obtained from cell phenotyping. For raw IMC images, the pretrained ResNet-50 model with weights pretrained on ImageNet is first utilized to extract embeddings from each channel within the multiplex IMC channels. Each channel is fed to the three-channels of ResNet-50 and the embeddings are computed before the classificationlayers are obtained. Each channel produces an embedding vector size of 2,048 and then these are all concatenated into a single vector of features representing that specific core. We then reduced the dimensionality of the extracted feature vectors using mini-batch sparse principal components analysis to a specific number of principal components (for most applications we tried nine principal components). Principal components were later used to train a support vector machine with a radial basis function kernel with the parameters specified in our codebase. For the imbalanced datasets, we used a random oversampling to achieve an equal number of samples for each class during the training. The function used is RandomOverSampler (version 0.9.1) and it is available at: https://imbalanced-learn.org/stable/references/generated/imblearn.over_sampling.RandomOverSampler.html. To compare with cell frequencies, we imagined that cell-frequency vectors also represent a core (in which each vector is simply a vector of cell prevalence of each type). Similar to images, we reduced the dimensionality of the extracted feature vectors to nine principal components and then trained a support vector machine with a radial basis function kernel with the same parameters. Various classes of Scikit-learn (version 1.0.2) machine-learning libraries have been utilized for the tasks of splitting the dataset, dimensionality reduction and training support vector machines for the prediction tasks. All feature extraction and training steps were performed on Google Cloud GPU/TPU servers. See the ‘Code availability’ section for additional details.

### Statistical analysis and workflow

All image analysis steps were performed in MATLAB (version 2019b) and Python (version 3.7.12). Statistical analyses were performed using RStudio version 4.2.2 and GraphPad Prism 9 statistical software. Data are expressed as mean ± s.e.m. or mean ± s.d.; *P* < 0.05 was considered significant unless otherwise indicated. All statistical tests are indicated in the figure legends. Survival data were analysed by log-rank (Mantel–Cox) test.

### Reporting summary

Further information on research design is available in the Nature Portfolio Reporting Summary linked to this article.

## Online content

Any methods, additional references, Nature Portfolio reporting summaries, source data, extended data, supplementary information, acknowledgements, peer review information; details of author contributions and competing interests; and statements of data and code availability are available at 10.1038/s41586-022-05672-3.

## Supplementary information

Supplementary Information This file contains Supplementary Data 1–2 and Supplementary Tables 1–15. The Supplementary Data describes single cell prevalence across clinical variables and the association of cellular neighborhood prevalence with survival across the five predominant histological patterns of lung adenocarcinoma. The Supplementary Tables describe the clinical information and summarize statistical analyses.

Reporting Summary

## Data Availability

Data supporting the findings in this study, including high-dimensional TIFF images, are available at 10.5281/zenodo.7383627. Raw primary imaging data can be obtained from the authors directly on reasonable request.

## Code availability

The original code used to produce the results of this study is available at https://github.com/walsh-quail-labs/IMC-Lung.

## References

[1] Leader AM (2021). Single-cell analysis of human non-small cell lung cancer lesions refines tumor classification and patient stratification. Cancer Cell.

[2] Melms JC (2021). A molecular single-cell lung atlas of lethal COVID-19. Nature.

[3] Marjanovic ND (2020). Emergence of a high-plasticity cell state during lung cancer evolution. Cancer Cell.

[4] Zilionis R (2019). Single-cell transcriptomics of human and mouse lung cancers reveals conserved myeloid populations across individuals and species. Immunity.

[5] Liu B (2022). Temporal single-cell tracing reveals clonal revival and expansion of precursor exhausted T cells during anti-PD-1 therapy in lung cancer. Nat. Cancer.

[6] Zheng L (2021). Pan-cancer single-cell landscape of tumor-infiltrating T cells. Science.

[7] Cheng S (2021). A pan-cancer single-cell transcriptional atlas of tumor infiltrating myeloid cells. Cell.

[8] Kumar V (2022). Single-cell atlas of lineage states, tumor microenvironment, and subtype-specific expression programs in gastric cancer. Cancer Discov..

[9] Zhang Y (2021). Single-cell analyses of renal cell cancers reveal insights into tumor microenvironment, cell of origin, and therapy response. Proc. Natl Acad. Sci. USA.

[10] Siegel RL, Miller KD, Fuchs HE, Jemal A (2021). Cancer statistics, 2021. CA Cancer J. Clin..

[11] Gridelli C (2015). Non-small-cell lung cancer. Nat. Rev. Dis. Primers.

[12] Jackson HW (2020). The single-cell pathology landscape of breast cancer. Nature.

[13] Ali HR (2020). Imaging mass cytometry and multiplatform genomics define the phenogenomic landscape of breast cancer. Nat. Cancer.

[14] Schurch CM (2020). Coordinated cellular neighborhoods orchestrate antitumoral immunity at the colorectal cancer invasive front. Cell.

[15] Tavernari D (2021). Nongenetic evolution drives lung adenocarcinoma spatial heterogeneity and progression. Cancer Discov..

[16] Casanova-Acebes M (2021). Tissue-resident macrophages provide a pro-tumorigenic niche to early NSCLC cells. Nature.

[17] Welsh TJ (2005). Macrophage and mast-cell invasion of tumor cell islets confers a marked survival advantage in non-small-cell lung cancer. J. Clin. Oncol..

[18] Wu P (2016). Inverse role of distinct subsets and distribution of macrophage in lung cancer prognosis: a meta-analysis. Oncotarget.

[19] Conforti F (2021). Sex-based dimorphism of anticancer immune response and molecular mechanisms of immune evasion. Clin. Cancer Res..

[20] Tong BC (2014). Sex differences in early outcomes after lung cancer resection: analysis of the Society of Thoracic Surgeons General Thoracic Database. J. Thorac. Cardiovasc. Surg..

[21] International Early Lung Cancer Action Program Investigators. (2006). Women’s susceptibility to tobacco carcinogens and survival after diagnosis of lung cancer. JAMA.

[22] Weng NP (2006). Aging of the immune system: how much can the adaptive immune system adapt?. Immunity.

[23] Kugel CH (2018). Age correlates with response to anti-PD1, reflecting age-related differences in intratumoral effector and regulatory T-cell populations. Clin. Cancer Res..

[24] Fane M, Weeraratna AT (2020). How the ageing microenvironment influences tumour progression. Nat. Rev. Cancer.

[25] Yager EJ (2008). Age-associated decline in T cell repertoire diversity leads to holes in the repertoire and impaired immunity to influenza virus. J. Exp. Med..

[26] Szczerba BM (2019). Neutrophils escort circulating tumour cells to enable cell cycle progression. Nature.

[27] Saini M, Szczerba BM, Aceto N (2019). Circulating tumor cell–neutrophil tango along the metastatic process. Cancer Res..

[28] Xu L, Tavora F, Burke A (2013). Histologic features associated with metastatic potential in invasive adenocarcinomas of the lung. Am. J. Surg. Pathol..

[29] Enfield KSS (2019). Hyperspectral cell sociology reveals spatial tumor-immune cell interactions associated with lung cancer recurrence. J. Immunother. Cancer.

[30] Peranzoni E (2018). Macrophages impede CD8 T cells from reaching tumor cells and limit the efficacy of anti-PD-1 treatment. Proc. Natl Acad. Sci. USA.

[31] Schultze JL (1997). CD40-activated human B cells: an alternative source of highly efficient antigen presenting cells to generate autologous antigen-specific T cells for adoptive immunotherapy. J. Clin. Invest..

[32] Folkman J (1971). Tumor angiogenesis: therapeutic implications. N. Engl. J. Med..

[33] Noman MZ (2014). PD-L1 is a novel direct target of HIF-1α, and its blockade under hypoxia enhanced MDSC-mediated T cell activation. J. Exp. Med..

[34] Chang CF (2012). Polar opposites: Erk direction of CD4 T cell subsets. J. Immunol..

[35] He, K., Zhang, X., Ren, S. & Sun, J. Deep residual learning for image recognition. In *2016 IEEE Conference on Computer Vision and Pattern Recognition (CVPR)* 770–778 (IEEE, 2016).

[36] Deng, J. et al. ImageNet: a large-scale hierarchical image database. In *2009 IEEE Conference on Computer Vision and Pattern Recognition* 248–255 (IEEE, 2009).

[37] Ettinger DS (2022). Non-small cell lung cancer, version 3.2022, NCCN Clinical Practice Guidelines in Oncology. J. Natl Compr. Canc. Netw..

[38] Pisters K (2007). Cancer Care Ontario and American Society of Clinical Oncology adjuvant chemotherapy and adjuvant radiation therapy for stages I–IIIA resectable non small-cell lung cancer guideline. J. Clin. Oncol..

[39] Martini N (1995). Incidence of local recurrence and second primary tumors in resected stage I lung cancer. J. Thorac. Cardiovasc. Surg..

[40] Felip E (2021). Adjuvant atezolizumab after adjuvant chemotherapy in resected stage IB–IIIA non-small-cell lung cancer (IMpower010): a randomised, multicentre, open-label, phase 3 trial. Lancet.

[41] Wu YL (2020). Osimertinib in resected EGFR-mutated non-small-cell lung cancer. N. Engl. J. Med..

[42] Liudahl SM (2021). Leukocyte heterogeneity in pancreatic ductal adenocarcinoma: phenotypic and spatial features associated with clinical outcome. Cancer Discov..

[43] Peng H (2021). Profiling tumor immune microenvironment of non-small cell lung cancer using multiplex immunofluorescence. Front. Immunol..

[44] Ginhoux F, Schultze JL, Murray PJ, Ochando J, Biswas SK (2016). New insights into the multidimensional concept of macrophage ontogeny, activation and function. Nat. Immunol..

[45] Murray PJ (2014). Macrophage activation and polarization: nomenclature and experimental guidelines. Immunity.

[46] Vassilvitskii, S. & Arthur, D. in *SODA '07:**Proc. 18th Annual ACM–SIAM Symposium on Discrete Algorithms*, 1027–1035 (Society for Industrial and Applied Mathematics, 2007).

[47] MacLahlan, G. & Peel, D. *Finite Mixture Models* (John Wiley & Sons, 2000).

