# Supplementary material for: Single-cell spatial landscapes of the lung tumour immune microenvironment
Source: Nature. 2023 Feb 1;614(7948):548–54. doi: 10.1038/s41586-022-05672-3 (PMC9931585; doi:10.1038/s41586-022-05672-3)
Supplement: Supplementary file 1 — This file contains Supplementary Data 1–2 and Supplementary Tables 1–15. The Supplementary Data describes single cell prevalence across clinical variables and the association of cellular neighborhood prevalence with survival across the five predominant histological patterns of lung adenocarcinoma. The Supplementary Tables describe the clinical information and summarize statistical analyses. [file 41586_2022_5672_MOESM1_ESM.pdf]

nature portfolio

https://doi.org/10.1038/s41586-022-05672-3

Supplementary information

Single-cell spatial landscapes of the lung tumour immune microenvironment

In the format provided by the authors and unedited

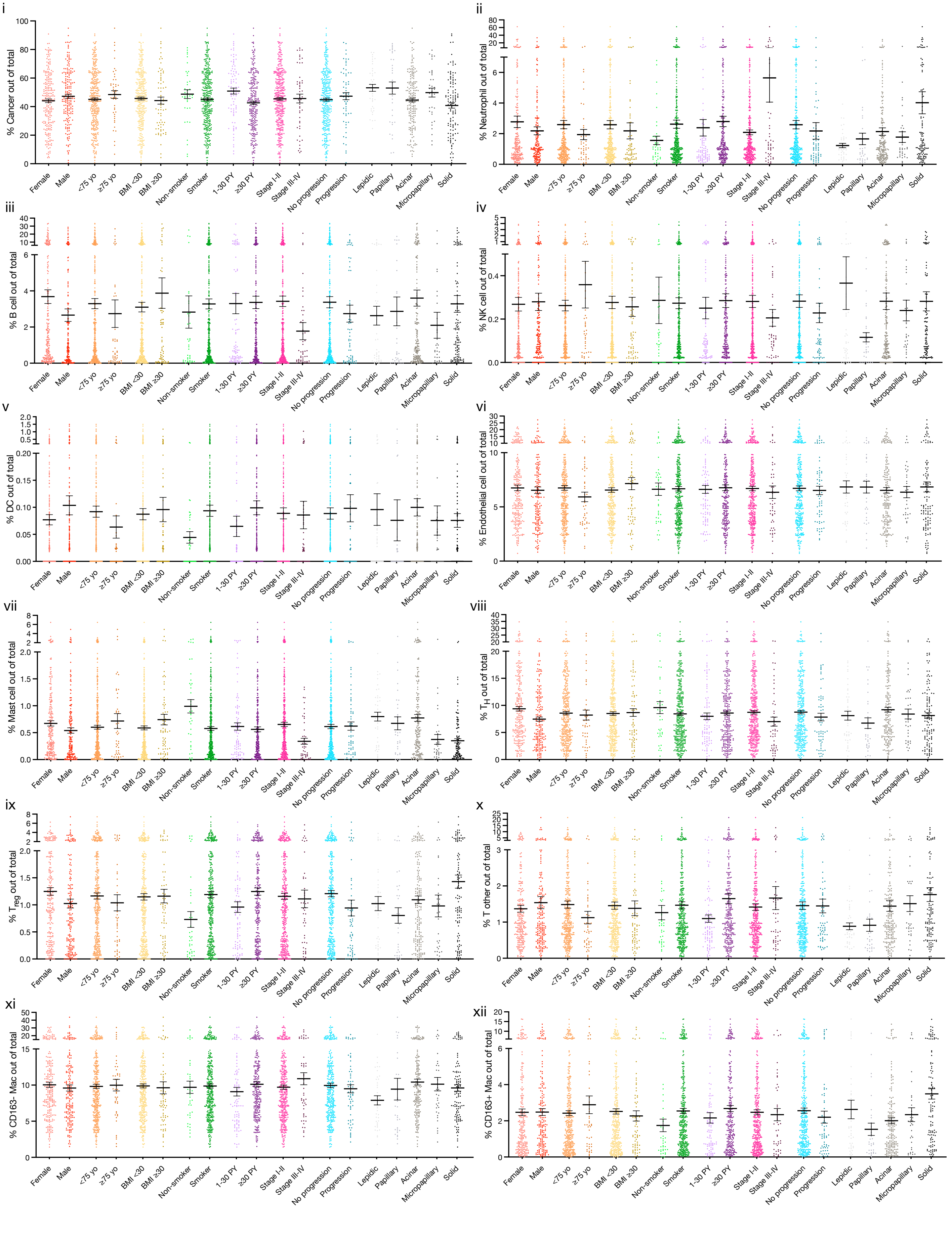
i
% Cancer out of total
ii
% Neutrophil out of total
iii
% B cell out of total
iv
% NK cell out of total
v
% DC out of total
vi
% Endothelial cell out of total
vii
% Mast cell out of total
viii
% T_H out of total
ix
% T_{reg} out of total
x
% T other out of total
xi
% CD163- Mac out of total
xii
% CD163+ Mac out of total
Female
Male
<75 yo
≥75 yo
BMI <30
BMI ≥30
Non-smoker
Smoker
1-30 PY
≥30 PY
Stage I-II
Stage III-IV
No progression
Progression
Lepidic
Papillary
Acinar
Micropapillary
Solid

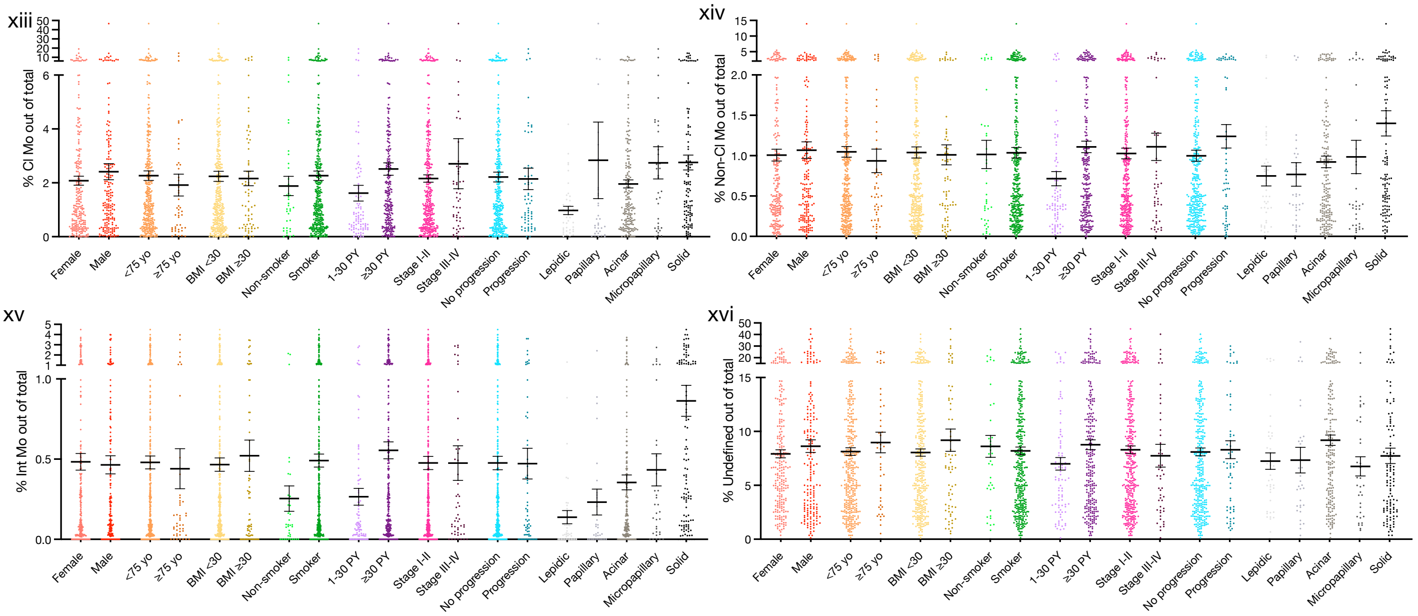

Supplementary Fig. 1: Single cell prevalence across clinical variables. Prevalence of cancer cells, neutrophils, B cells, NK cells, DC cells, endothelial cells, mast cells, T_H, T_{reg}, T other, M1-like Mac, M2-like Mac, Cl Mo, Int Mo, Non-Cl Mo and undefined cells across sex (Female n = 233, Male n = 183), age (<75 yo n = 369, ≥75 yo n = 47), BMI (<30 n = 346, ≥30 n = 70), smoking status (Smoker n = 376, Non-smoker n = 38), pack-years (1-30 n = 89, ≥30 n = 256), stage (I-II n = 365, III-IV n = 50), progression status (Progression n = 64, No progression n = 340), and histological subgroup (Lepidic n = 40, Papillary n = 33, Acinar n = 190, Micropapillary n = 35, Solid n = 118). For exact P values, see Supplementary Table 4. Mean ± SEM.

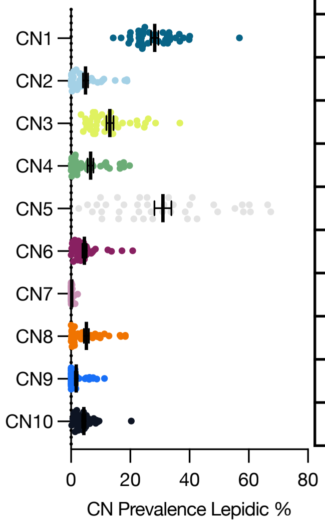

k = 10 Lepidic	P value (Log-rank test)
CN1	0.9361
CN2	0.1048
CN3	0.7888
CN4	0.02131
CN5	0.5508
CN6	0.5687
CN7	0.6787
CN8	0.1886
CN9	0.1377
CN10	0.8729

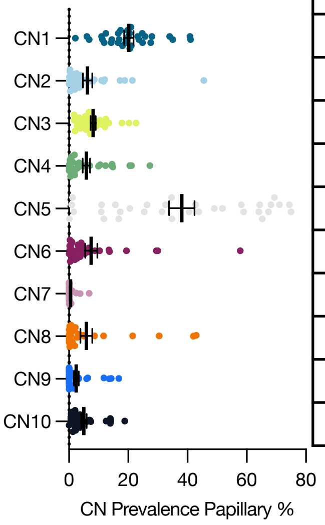

k = 10 Papillary	P value (Log-rank test)
CN1	0.9492
CN2	0.2331
CN3	0.3526
CN4	0.2182
CN5	0.7340
CN6	0.2797
CN7	0.7492
CN8	0.2089
CN9	0.4532
CN10	0.3315

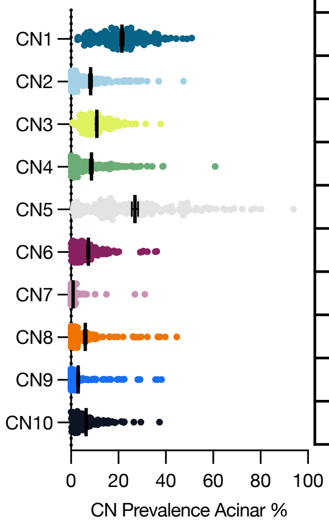

k = 10 Acinar	P value (Log-rank test)
CN1	0.8178
CN2	0.1526
CN3	0.09386
CN4	0.3959
CN5	0.9222
CN6	0.3456
CN7	0.1482
CN8	0.4684
CN9	0.0329
CN10	0.2879

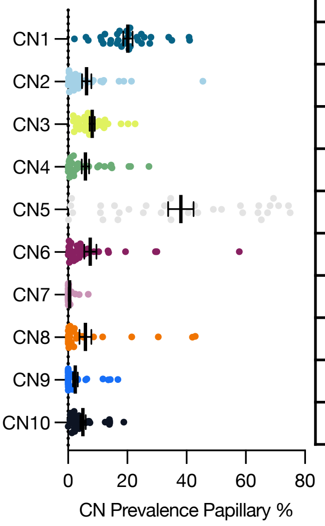

k = 10 Micropapillary	P value (Log-rank test)
CN1	0.9832
CN2	0.4658
CN3	0.6584
CN4	0.2461
CN5	0.2297
CN6	0.7798
CN7	0.05359
CN8	0.1284
CN9	0.1459
CN10	0.2003

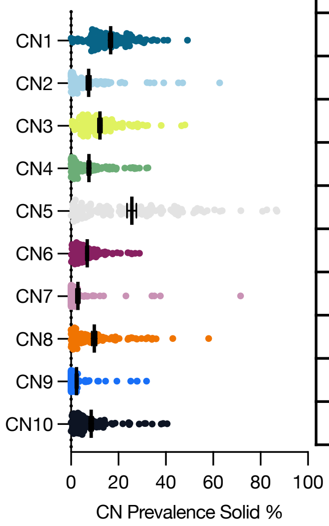

k = 10 Solid	P value (Log-rank test)
CN1	0.2926
CN2	0.02125
CN3	0.3408
CN4	0.009689
CN5	0.8473
CN6	0.2238
CN7	0.6852
CN8	0.2774
CN9	0.1329
CN10	0.1053

Supplementary Fig. 2: Cellular neighbourhood prevalence across 5 major histological patterns in 416 lung adenocarcinoma patients. Prevalence of 10 cellular neighbourhoods across the five histological subtypes (Lepidic n = 40, Papillary n = 33, Acinar n = 190, Micropapillary n = 35, Solid n = 118). Tables depict result of log-rank test for the overall survival based on low (z score <0) and high (z score ≥0) prevalence of depicted neighbourhoods across histological subtypes. Black p values indicate no significant difference ($p \geq 0.05$) in survival between the two groups. Blue p values indicate better survival ($p < 0.05$) with high prevalence and red p values indicate better survival ($p < 0.05$) with low prevalence of the depicted groups. Mean ± SEM. Statistical analysis (log-rank test).

	Count	Percentage
**Sex**		
Female	233	56.01%
Male	183	43.99%
**Age**		
<75	369	88.70%
≥75	47	11.30%
**BMI**		
<30	346	83.17%
≥30	70	16.83%
**Smoking Status**		
Smoker	376	90.38%
Non-smoker	38	9.13%
NA	2	0.48%
**Pack Years**		
1-30	89	21.39%
≥30	256	61.54%
NA	71	17.07%
**Stage**		
I-II	365	87.74%
III-IV	50	12.02%
NA	1	0.24%
**Progression**		
Yes	64	15.38%
No	340	81.73%
NA	12	2.88%
**Survival**		
<3 years	89	21.39%
≥3 years	318	76.44%
NA	9	2.16%
**Histology**		
Lepidic	40	9.62%
Papillary	33	7.93%
Acinar	190	45.67%
Micropapillary	35	8.41%
Solid	118	28.37%

Supplementary Table 1: Clinical characteristics of NSCLC cohort.

Metal	Marker	Clone	Dilution	Catalog #	Supplier
141Pr	αSMA	1A4	1:100	3141017D	Fluidigm
142Nd	CD16	SP175	1:100	ab183354	Abcam
143Nd	Ki-67	B56	1:100	ab279657	Abcam
144Nd	CD14	SP192	1:100	ab183322	Abcam
145Nd	MPO	EPR20257	1:100	ab208670	Abcam
146Nd	BCL2	EPR17509	1:100	3146019D	Fluidigm
147Sm	CD31	EP3095	1:100	ab216459	Abcam
148Nd	TTF1	SP141	1:100	ab242428	Abcam
149Sm	HIF1α	EP1215Y	1:100	ab51608	Abcam
150Nd	PD-L1/Biotin	E1L3N/1D4-C5	1:50/1:50	13684T/3150008B	Cell Signaling Technologies/Fluidigm
151Eu	M-CSF-R	SP211	1:100	ab183316	Abcam
152Sm	CD45	D9M8I	1:100	3152018D	Fluidigm
153Eu	CD117	YR145	1:100	ab32363	Abcam
154Sm	CD11c	EP1347Y	1:100	ab52632	Abcam
155Gd	FOXP3	236A/E7	1:50	ab20034	Abcam
156Gd	CD4	EPR6855	1:100	ab133616	Abcam
158Gd	ARG1	EPR6672(B)	1:100	ab211961	Abcam
159Tb	CD68	KP1	1:50	3159035D	Fluidigm
160Gd	MMP9	EP1255Y	1:100	ab137867	Abcam
161Dy	CD20	H1	1:100	3161029D	Fluidigm
162Dy	CD8α	C8/144B	1:50	3162034D	Fluidigm
163Dy	pSTAT3	EP2147Y	1:100	ab76315	Abcam
164Dy	CD163	EPR19518	1:100	ab213612	Abcam
165Ho	PD1	EPR4877(2)	1:50	3165039D	Standard BioTools
166Er	B7-H4	H74	1:100	3166030D	Standard BioTools
167Er	CD39	EPR20627	1:100	ab223842	Abcam
168Er	CD40	EPR20540	1:50	ab213205	Abcam
169Tm	CD94	EPR21003	1:100	ab238166	Abcam
170Er	CD3	Polyclonal	1:50	3170019D	Fluidigm
171Yb	pERK	D13.14.4E	1:100	3171010A	Fluidigm
172Yb	CC3	5A1E	1:100	3171021D	Standard BioTools
173Yb	B7-H3	3173014D	1:100	3172027D	Standard BioTools
174Yb	HLA-DR	EPR3692	1:100	ab92511	Abcam
175Lu	PanCK	AE1+AE3	1:100	ab80826	Abcam
176Yb	Histone H3	DIH2	1:100	3176023D	Fluidigm

Supplementary Table 2: Optimized antibody panel for imaging mass cytometry.

Histological comparison	P value (Log-rank test)
Lepidic vs Papillary	0.0078
Lepidic vs Acinar	0.0095
Lepidic vs Micropapillary	0.0143
Lepidic vs Solid	0.0002
Papillary vs Acinar	0.5761
Papillary vs Micropapillary	0.9398
Papillary vs Solid	0.3289
Acinar vs Micropapillary	0.5446
Acinar vs Solid	0.0099
Micropapillary vs Solid	0.3856

Supplementary Table 3: Log-rank p-values comparing survival in five histological subtypes. Colour of the p values indicates worse overall survival for the histological group of the same colour.

	Cancer	B Cell	Neutrophil	NK Cell	Dendritic Cell	Endothelial Cell	Mast Cell	T_c	T_H	T_{reg}	T other	CD163- Mac	CD163+ Mac	Cl Mo	Non-Cl Mo	Int Mo	Undefined
Female vs male	0.342	0.181	0.527	0.880	0.378	0.888	0.148	0.932	0.006	0.087	0.646	0.855	0.965	0.702	0.924	0.993	0.679
<75 y/o vs ≥75 y/o	0.476	0.702	0.547	0.880	0.594	0.888	0.427	0.066	0.816	0.640	0.646	0.855	0.566	0.702	0.924	0.993	0.750
BMI <30 vs BMI ≥30	0.647	0.627	0.547	0.880	0.855	0.888	0.182	0.707	0.816	0.924	0.889	0.855	0.666	0.878	0.925	0.993	0.679
Non-smoker vs smoker	0.476	0.725	0.527	0.880	0.378	0.945	0.005	0.066	0.452	0.087	0.722	0.855	0.431	0.702	0.925	0.245	0.821
1-30 PY vs ≥30 PY	0.005	0.921	0.547	0.880	0.378	0.888	0.639	0.066	0.573	0.087	0.154	0.564	0.431	0.259	0.021	0.017	0.262
Stage I-II vs Stage III-IV	0.938	0.181	8.24E-06	0.880	0.916	0.888	0.013	0.253	0.178	0.902	0.646	0.564	0.866	0.702	0.924	0.993	0.821
No progression vs progression	0.521	0.686	0.547	0.880	0.855	0.888	0.939	0.570	0.452	0.132	0.978	0.855	0.566	0.878	0.558	0.993	0.821
Low vs high expression (survival)*	0.908	0.001	0.357	0.745	0.659	0.362	0.050	0.653	0.061	0.314	0.517	0.420	0.004	0.560	0.050	0.010	0.068
Lepidic vs Papillary	1	1	0.996	0.222	0.992	1	0.940	0.848	0.855	0.909	1	0.758	0.371	0.114	1	0.983	1
Lepidic vs Acinar	0.078	0.834	0.814	0.876	1	0.991	1	1	0.817	0.996	0.400	0.069	0.635	0.420	0.926	0.448	0.471
Lepidic vs Micropapillary	0.945	0.993	0.988	0.818	0.991	0.986	0.066	0.999	0.999	1	0.574	0.411	0.989	0.139	0.921	0.423	0.998
Lepidic vs Solid	0.004	0.963	0.015	0.895	0.979	1	0.004	0.136	1	0.240	0.068	0.439	0.359	0.026	0.031	1.46E-06	0.995
Papillary vs Acinar	0.137	0.950	0.984	0.410	0.964	0.994	0.942	0.599	0.166	0.609	0.541	0.884	0.867	0.616	0.963	0.905	0.597
Papillary vs Micropapillary	0.960	0.976	1	0.852	1	0.989	0.392	0.953	0.761	0.962	0.662	0.987	0.696	1	0.951	0.797	0.997
Papillary vs Solid	0.011	0.995	0.096	0.456	1	1	0.128	0.007	0.740	0.028	0.130	1	0.001	1	0.069	0.00019	0.998
Acinar vs Micropapillary	0.570	0.545	0.994	0.991	0.959	0.999	0.017	0.983	0.947	0.978	1	0.999	0.955	0.693	0.999	0.979	0.293
Acinar vs Solid	0.439	0.986	0.009	1	0.820	0.962	3.30E-06	0.008	0.524	0.063	0.571	0.727	1.27E-05	0.235	0.008	9.64E-08	0.359
Micropapillary vs Solid	0.103	0.780	0.114	0.993	1	0.972	1	0.080	0.999	0.194	0.954	0.989	0.142	1	0.396	0.023	0.945

Supplementary Table 4: Comparison of cell frequencies between various clinical parameters.
Colour of the p values indicates higher prevalence or improved survival* for the clinical group of the same colour. FDR-corrected two-tailed Student t-test for sex, age, BMI, smoking status, pack-years, stage, progression status; one-way ANOVA with Tukey multiple comparison test for histological group; log-rank test for survival.

	Hazard Ratio	95% CI for Hazard Ratio		Significance (p value)
		Lower	Upper	
pERK+ T_H	0.949	0.914	0.985	0.005
HIF1α+ neutrophils	1.006	1.0001	1.011	0.046
Ki-67+ endothelial cells	1.084	1.009	1.163	0.027

Supplementary Table 5: Univariate Cox regression of the association between the prevalence of pERK+ T_H, HIF-1a+ neutrophils and Ki-67+ endothelial cells and survival outcomes.

	CN1	CN2	CN3	CN4	CN5	CN6	CN7	CN8	CN9	CN10
Female vs male	0.806	0.338	0.820	0.015	0.304	0.765	0.665	0.907	0.287	0.678
<75 y/o vs ≥75 y/o	0.816	0.806	0.585	0.960	0.304	0.987	0.665	0.302	0.908	0.496
BMI <30 vs BMI ≥30	0.316	0.338	0.219	0.960	0.913	0.987	0.834	0.907	0.287	0.496
Non-smoker vs smoker	0.015	0.887	0.955	0.425	0.913	0.987	0.665	0.078	0.908	0.430
1-30 PY vs ≥30 PY	0.720	0.187	0.463	0.425	0.004	0.765	0.897	0.078	0.908	0.496
Stage I-II vs Stage III-IV	0.013	0.887	0.078	0.425	0.318	0.230	1.57E-06	0.907	0.287	0.874
No progression vs progression	0.828	0.887	0.621	0.425	0.430	0.765	0.834	0.404	0.595	0.496
Low vs high expression (survival)*	0.375	0.006	0.026	0.039	0.888	0.473	0.100	0.797	0.001	0.893
Lepidic vs Papillary	0.002	0.965	0.019	0.996	0.550	0.356	0.999	0.998	0.989	0.990
Lepidic vs Acinar	0.00032	0.194	0.333	0.616	0.757	0.136	0.934	0.984	0.751	0.359
Lepidic vs Micropapillary	0.004	0.996	0.713	0.910	0.981	0.473	0.999	0.998	0.998	0.884
Lepidic vs Solid	2.19E-10	0.520	0.955	0.964	0.567	0.388	0.048	0.039	0.982	0.004
Papillary vs Acinar	0.936	0.764	0.222	0.402	0.024	1	0.994	1	0.987	0.807
Papillary vs Micropapillary	0.999	0.999	0.393	0.767	0.879	1	1	1	0.941	0.993
Papillary vs Solid	0.305	0.963	0.023	0.834	0.013	0.983	0.160	0.161	1	0.056
Acinar vs Micropapillary	0.990	0.507	1	0.999	0.362	1	0.995	1	0.548	0.981
Acinar vs Solid	8.83E-05	0.938	0.448	0.824	0.980	0.958	0.016	0.003	0.886	0.046
Micropapillary vs Solid	0.150	0.834	0.915	0.994	0.228	0.999	0.155	0.138	0.901	0.176

Supplementary Table 6: Comparison of ten cellular neighbourhoods between various clinical parameters. Colour of the p values indicates higher prevalence or improved survival* for the clinical group of the same colour. FDR-corrected two-tailed Student t-test for sex, age, BMI, smoking status, pack-years, stage, progression status; one-way ANOVA with Tukey multiple comparison test for histological group; log-rank test for survival.

	CN1	CN2	CN3	CN4	CN5	CN6	CN7	CN8	CN9	CN10	CN11	CN12	CN13	CN14	CN15
Female vs male	0.167	0.646	0.854	0.407	0.625	0.955	0.262	0.699	0.608	0.222	0.337	0.637	0.945	0.841	0.487
<75 y/o vs ≥75 y/o	0.819	0.719	0.501	0.904	0.930	0.955	0.622	0.699	0.608	0.222	0.919	0.637	0.537	0.663	0.687
BMI <30 vs BMI ≥30	0.167	0.719	0.854	0.475	0.868	0.955	0.622	0.418	0.878	0.700	0.056	0.637	0.537	0.841	0.487
Non-smoker vs smoker	0.888	0.608	0.020	0.904	0.625	0.435	0.262	0.858	0.608	0.700	0.919	0.637	0.472	0.663	0.007
1-30 PY vs ≥30 PY	0.167	0.209	0.466	0.407	0.614	0.955	0.262	0.903	0.894	0.002	0.919	0.637	0.537	0.663	0.487
Stage I-II vs Stage III-IV	0.888	0.601	0.466	0.904	0.016	0.955	0.262	0.903	2.14E-06	0.222	0.337	0.897	0.793	0.663	0.003
No progression vs progression	0.888	0.788	0.466	0.904	0.868	0.955	0.866	0.953	0.878	0.540	0.640	0.637	0.487	0.959	0.487
Low vs high expression (survival)*	0.003	0.837	0.163	0.349	0.139	0.429	0.093	0.459	0.215	0.522	0.039	0.048	0.697	0.412	0.069
Lepidic vs Papillary	0.946	0.964	0.339	0.982	0.416	0.977	0.997	0.879	0.999	0.178	0.970	0.133	0.943	0.512	0.007
Lepidic vs Acinar	0.362	0.00048	0.001	0.598	0.009	0.043	0.990	0.989	0.934	0.991	0.781	0.116	0.591	0.994	0.023
Lepidic vs Micropapillary	1	0.457	0.097	0.771	0.227	0.881	0.938	0.988	0.999	0.861	1	0.626	0.986	0.394	0.024
Lepidic vs Solid	0.442	9.99E-06	4.18E-06	0.738	0.015	0.067	0.498	0.896	0.054	1	0.999	1	0.014	0.037	1.27E-06
Papillary vs Acinar	0.943	0.027	0.687	0.257	0.897	0.350	0.917	0.484	0.995	0.014	0.999	0.955	0.995	0.505	0.607
Papillary vs Micropapillary	0.984	0.880	0.980	0.467	0.998	0.998	0.994	0.636	1	0.752	0.945	0.884	0.999	1	0.994
Papillary vs Solid	0.960	0.001	0.082	0.377	0.911	0.409	0.329	0.998	0.180	0.062	0.982	0.027	0.249	0.941	0.875
Acinar vs Micropapillary	0.581	0.385	0.975	1	0.987	0.590	0.617	1	0.995	0.449	0.714	0.986	0.950	0.355	0.888
Acinar vs Solid	1	0.457	0.188	0.999	1	1	0.373	0.215	0.020	0.967	0.658	0.001	0.044	0.001	0.001
Micropapillary vs Solid	0.650	0.053	0.318	0.999	0.989	0.647	0.104	0.596	0.169	0.746	0.995	0.344	0.119	0.977	0.563

	CN16	CN17	CN18	CN19	CN20	CN21	CN22	CN23	CN24	CN25	CN26	CN27	CN28	CN29	CN30
Female vs male	0.017	0.880	0.905	0.767	0.456	0.052	0.347	0.964	0.694	0.434	0.796	0.411	0.953	0.081	0.410
<75 y/o vs ≥75 y/o	0.878	0.880	0.271	0.486	0.161	0.862	0.347	0.964	0.694	0.991	0.869	0.168	0.264	0.451	0.410
BMI <30 vs BMI ≥30	0.902	0.880	0.905	0.767	0.213	0.847	0.347	0.964	0.694	0.991	0.796	0.255	0.953	0.782	0.410
Non-smoker vs smoker	0.878	0.880	0.141	0.486	0.213	0.238	0.347	0.604	0.694	0.991	0.796	0.255	0.025	0.346	0.519
1-30 PY vs ≥30 PY	0.047	0.880	0.145	0.486	0.053	0.984	0.347	0.173	0.694	0.991	0.796	0.181	0.953	0.451	0.410
Stage I-II vs Stage III-IV	0.878	0.880	0.782	0.779	0.053	0.563	0.958	0.964	0.694	0.434	0.975	0.168	0.264	0.081	0.410
No progression vs progression	0.878	0.880	0.145	0.727	0.856	0.563	0.506	0.964	0.929	0.745	0.796	0.812	0.352	0.451	0.554
Low vs high expression (survival)*	0.936	0.752	0.265	0.287	0.61	0.076	0.139	0.00042	0.688	0.003	0.281	0.805	0.350	0.031	0.142
Lepidic vs Papillary	0.966	1	0.997	0.898	0.264	0.992	0.999	0.993	0.069	0.994	0.613	0.141	0.346	0.043	0.991
Lepidic vs Acinar	0.539	0.703	0.921	1	0.628	0.625	0.509	0.050	0.864	0.754	0.777	0.548	0.038	0.531	0.997
Lepidic vs Micropapillary	0.436	0.958	0.997	0.999	0.751	0.997	0.585	0.638	0.413	1	0.900	0.591	0.023	0.418	0.263
Lepidic vs Solid	0.302	0.830	0.008	0.995	0.624	1	0.002	0.106	0.294	0.982	0.998	1	5.77E-08	0.040	0.979
Papillary vs Acinar	0.170	0.867	0.998	0.859	0.744	0.944	0.769	0.271	0.129	0.975	0.954	0.573	0.998	0.239	0.999
Papillary vs Micropapillary	0.163	0.987	1	0.964	0.93	1	0.767	0.894	0.903	0.980	0.984	0.913	0.816	0.825	0.581
Papillary vs Solid	0.077	0.937	0.060	0.607	0.002	0.980	0.014	0.397	0.681	1	0.630	0.027	0.009	0.952	0.816
Acinar vs Micropapillary	0.960	0.997	0.996	1	1	0.899	0.997	0.916	0.718	0.651	1	0.995	0.803	0.954	0.180
Acinar vs Solid	0.948	0.999	0.001	0.927	0.00023	0.196	0.006	0.999	0.508	0.891	0.748	0.078	6.01E-06	0.219	0.625
Micropapillary vs Solid	1	1	0.047	0.970	0.045	0.991	0.393	0.965	0.999	0.945	0.941	0.297	0.254	0.974	0.027

Supplementary Table 7: Comparison of 30 cellular neighbourhoods between various clinical parameters.
Colour of the p values indicates higher prevalence or improved survival* for the clinical group of the same colour. FDR-corrected two-tailed Student t-test for sex, age, BMI, smoking status, pack-years, stage, progression status; one-way ANOVA with Tukey multiple comparison test for histological group; log-rank test for survival

	Baseline	Our Score 5-Fold Cross Validation					Mean	Standard Deviation
		Fold #1	Fold #2	Fold #3	Fold #4	Fold #5		
Histological Type	45.67%	43.33%	49.12%	50.00%	50.00%	42.59%	47.01%	3.72%
Sex - Male/Female	56.00%	60.24%	44.57%	54.21%	54.21%	50.60%	52.77%	5.74%
Survival 3 Years	76.44%	74.66%	70.00%	78.12%	81.81%	75.75%	76.07%	4.36%
BMI ≥30	83.17%	82.89%	94.36%	88.73%	89.55%	85.93%	88.29%	4.28%
Progression	84.15%	86.76%	93.33%	87.67%	85.07%	86.15%	87.80%	3.23%
Stage 1-2 vs 3-4	87.95%	90.90%	87.95%	97.46%	92.85%	91.17%	92.07%	3.49%
Age ≥75	88.70%	92.64%	93.67%	96.00%	95.77%	94.66%	94.55%	1.42%
Smoking	90.82%	88.31%	96.10%	91.55%	91.03%	97.26%	92.85%	3.73%

Supplementary Table 8: Prediction scores for a discovery cohort consisting of 416 NSCLC patients (416 cores) using cell frequencies.

	Baseline	Our Score 5-Fold Cross Validation					Mean	Standard Deviation
		Fold #1	Fold #2	Fold #3	Fold #4	Fold #5		
Histological Type	45.67%	64.17%	70.83%	59.25%	70.90%	60.78%	65.19%	5.48%
Sex - Male/Female	56.00%	45.23%	56.62%	39.75%	60.24%	60.24%	52.41%	9.38%
Survival 3 Years	76.44%	81.57%	91.93%	81.42%	92.18%	95.45%	88.51%	6.55%
BMI ≥30	83.17%	98.68%	94.36%	95.71%	98.48%	98.43%	97.13%	1.98%
Progression	84.15%	92.75%	98.64%	94.44%	100%	100%	97.17%	3.36%
Stage 1-2 vs 3-4	87.95%	100%	100%	96.00%	93.24%	100%	97.85%	3.10%
Age ≥75	88.70%	100%	100%	98.66%	100%	98.86%	99.50%	0.683%
Smoking	90.82%	100%	98.70%	100%	97.40%	98.63%	98.95%	1.09%

Supplementary Table 9: Prediction scores for a discovery cohort consisting of 416 NSCLC patients (416 cores) using raw images of lineage markers.

	Baseline	Our Score 5-Fold Cross Validation					Mean	Standard Deviation
		Fold #1	Fold #2	Fold #3	Fold #4	Fold #5		
Histological Type	45.67%	55.22%	75.00%	68.51%	65.45%	58.82%	64.60%	7.84%
Sex - Male/Female	56.00%	41.66%	60.24%	46.98%	53.01%	50.60%	50.50%	6.92%
Survival 3 Years	76.44%	86.84%	87.09%	80.00%	81.25%	90.90%	85.22%	4.51%
BMI ≥30	83.17%	100%	94.36%	94.28%	98.48%	95.31%	96.49%	2.60%
Progression	84.15%	96.29%	95.35%	96.66%	92.75%	98.64%	95.94%	2.15%
Stage 1-2 vs 3-4	87.95%	100%	98.68%	98.66%	91.89%	98.55%	97.56%	3.22%
Age ≥75	88.70%	98.41%	97.53%	100%	100%	98.68%	98.92%	1.07%
Smoking	90.82%	100%	98.70%	100%	97.40%	98.63%	98.95%	1.09%

Supplementary Table 10: Prediction scores for a discovery cohort consisting of 416 NSCLC patients (416 cores) using raw images of all markers.

	Baseline	Our Score 5-Fold Cross Validation					Mean	Standard Deviation
		Fold #1	Fold #2	Fold #3	Fold #4	Fold #5		
Clinical variables	85.87%	77.19%	84.21%	84.21%	76.79%	80.36%	80.55%	3.61%
Cell frequencies	85.31%	92.85%	92.15%	90.69%	86.00%	79.54%	88.25%	5.55%
Lineage markers	85.31%	92.85%	96.07%	93.02%	100%	97.72%	95.93%	3.07%
All markers	85.31%	96.42%	94.11%	93.02%	100%	93.18%	95.35%	2.93%

Supplementary Table 11: Prediction scores for progression in a discovery cohort consisting of 286 Stage IA-IB <4cm patients (286 cores).

	Count	Percentage
**Sex**		
Female	36	60.00%
Male	24	40.00%
**Age**		
<75	49	81.67%
≥75	11	18.33%
**Smoking Status**		
Smoker	53	88.33%
Non-smoker	7	11.67%
**Pack Years**		
1-30	13	21.67%
≥30	38	63.33%
NA	9	15.00%
**Stage**		
I	60	100.00%
**Progression**		
Yes	15	25.00%
No	45	75.00%
NA	0	0%
**Histology**		
Lepidic	13	21.67%
Papillary	3	5.00%
Acinar	34	56.67%
Micropapillary	0	0%
Solid	8	13.33%
NA	2	3.33%

Supplementary Table 12: Clinical characteristics of a validation cohort consisting of 60 Stage IA-IB <4cm patients (120 cores).

	Baseline	Accuracy	Precision	Recall	Sensitivity	Specificity
Cell frequencies	75.00%	74.17%	75.65%	96.67%	96.67%	6.67%
Lineage markers	75.00%	94.17%	95.60%	96.67%	96.67%	86.67%
All markers	75.00%	93.33%	95.56%	95.56%	95.56%	86.67%

Supplementary Table 13: Prediction scores and error measures for progression in a validation cohort consisting of 60 Stage IA-IB <4cm patients (120 cores).

	Baseline	Our Score<br>5-Fold Cross Validation					Mean	Standard Deviation
		Fold #1	Fold #2	Fold #3	Fold #4	Fold #5		
CD20	85.31%	85.71%	96.08%	88.37%	96.00%	93.18%	91.87%	3.80%
CD68	85.31%	87.76%	88.78%	89.80%	88.66%	89.69%	88.94%	0.68%
αSMA	85.31%	84.69%	81.63%	87.76%	90.72%	91.75%	87.31%	3.43%
CD14	85.31%	84.69%	85.71%	89.80%	89.69%	84.54%	86.89%	2.16%
CD8α	85.31%	83.67%	82.65%	90.82%	88.66%	86.60%	86.48%	2.77%
MMP9	85.31%	83.67%	82.65%	82.65%	91.75%	88.66%	85.88%	3.36%
PanCK	85.31%	85.71%	86.73%	81.63%	87.63%	87.63%	85.87%	2.04%
pERK	85.31%	85.71%	87.76%	87.76%	78.35%	88.66%	85.65%	3.45%
CD45	85.31%	81.63%	81.63%	87.76%	81.44%	90.72%	84.64%	3.54%
HLA-DR	85.31%	81.63%	81.63%	87.76%	83.51%	88.66%	84.64%	2.75%
CD3	85.31%	82.65%	82.65%	85.71%	84.54%	84.54%	84.02%	1.09%
pSTAT3	85.31%	85.71%	87.76%	84.69%	78.35%	82.47%	83.80%	2.93%
CD31	85.31%	81.63%	90.82%	88.78%	81.44%	76.29%	83.79%	4.84%
CD163	85.31%	82.65%	87.76%	80.61%	78.35%	87.63%	83.40%	3.43%
ARG1	85.31%	78.57%	85.71%	81.63%	85.57%	83.51%	83.00%	2.44%
CD11c	85.31%	76.53%	81.63%	87.76%	85.57%	81.44%	82.59%	3.52%
CD40	85.31%	78.57%	84.69%	89.80%	85.57%	74.23%	82.57%	5.02%
CD94	85.31%	85.71%	84.69%	81.63%	80.41%	79.38%	82.37%	2.23%
TTF1	85.31%	78.57%	77.55%	84.69%	85.57%	84.54%	82.18%	3.10%
CC3	85.31%	82.65%	88.78%	82.65%	77.32%	78.35%	81.95%	3.70%
CD4	85.31%	74.49%	83.67%	83.67%	80.41%	86.60%	81.77%	3.77%
CD16	85.31%	79.59%	80.61%	83.67%	81.44%	83.51%	81.77%	1.46%
HIF1α	85.31%	76.53%	83.67%	80.61%	83.51%	83.51%	81.57%	2.52%
BCL2	85.31%	80.61%	81.63%	84.69%	82.47%	76.29%	81.14%	2.53%
Histone H3	85.31%	79.59%	73.47%	86.73%	79.38%	81.44%	80.12%	3.89%
CD39	85.31%	76.53%	72.45%	83.67%	88.66%	78.35%	79.93%	5.17%
FOXP3	85.31%	79.59%	73.47%	82.65%	80.41%	80.41%	79.31%	2.82%
CD117	85.31%	77.55%	79.59%	78.57%	76.29%	79.38%	78.28%	1.12%
MPO	85.31%	74.49%	74.49%	73.47%	77.32%	80.41%	76.04%	2.32%
Ki-67	85.31%	75.51%	71.43%	79.59%	72.16%	79.38%	75.62%	3.15%
B7-H4	85.31%	72.45%	81.63%	76.53%	75.26%	71.13%	75.40%	3.34%

Supplementary Table 14: Prediction scores for progression in a discovery cohort of 286 Stage IA-IB <4cm patients (286 cores) using individual markers.

Description	Marker combination	Baseline	Accuracy	Precision	Recall
Top 2	CD20, CD68	75.00%	81.67%	85.56%	89.53%
Top 3	CD20, CD68, αSMA	75.00%	83.33%	85.56%	91.67%
Top 4	CD20, CD68, αSMA, CD14	75.00%	84.17%	88.89%	89.89%
Top 5	CD20, CD68, αSMA, CD14, CD8α	75.00%	85.83%	92.22%	89.25%
CN25	CD4, CD20	75.00%	85.00%	88.89%	90.91%
CN29	CD4, FOXP3	75.00%	85.00%	88.89%	90.91%
CN21	CD20, CD4, CD8, FOXP3	75.00%	85.83%	90.00%	91.01%
CN12	CD68, CD163, HLA-DR, CD11c, CD14, CD16	75.00%	89.17%	92.22%	93.26%
CN23	αSMA, CD117, CD94, CD16, CD14	75.00%	90.83%	93.33%	94.38%
CN23 + CD20	αSMA, CD117, CD94, CD16, CD14, CD20	75.00%	93.33%	95.55%	95.55%

Supplementary Table 15: Prediction scores and error measures for progression in a validation cohort consisting of 60 Stage IA-IB <4cm patients (120 cores) using marker combinations as indicated.
