# Supplementary material for: Single-cell spatial landscapes of the lung tumour immune microenvironment
Source: Nature. 2023 Feb 1;614(7948):548–54. doi: 10.1038/s41586-022-05672-3 (PMC9931585; doi:10.1038/s41586-022-05672-3)
Supplement: Supplementary file 2 — Reporting Summary [file 41586_2022_5672_MOESM2_ESM.pdf]

nature portfolio

Corresponding author(s): Daniela F. Quail, Philippe Joubert, Logan A. Walsh

Last updated by author(s): Dec 1, 2022

Reporting Summary

Nature Portfolio wishes to improve the reproducibility of the work that we publish. This form provides structure for consistency and transparency in reporting. For further information on Nature Portfolio policies, see our Editorial Policies and the Editorial Policy Checklist.

Statistics

For all statistical analyses, confirm that the following items are present in the figure legend, table legend, main text, or Methods section.

n/a	Confirmed	
☐	☒	The exact sample size (*n*) for each experimental group/condition, given as a discrete number and unit of measurement
☐	☒	A statement on whether measurements were taken from distinct samples or whether the same sample was measured repeatedly
☐	☒	The statistical test(s) used AND whether they are one- or two-sided<br>*Only common tests should be described solely by name; describe more complex techniques in the Methods section.*
☒	☐	A description of all covariates tested
☐	☒	A description of any assumptions or corrections, such as tests of normality and adjustment for multiple comparisons
☐	☒	A full description of the statistical parameters including central tendency (e.g. means) or other basic estimates (e.g. regression coefficient) AND variation (e.g. standard deviation) or associated estimates of uncertainty (e.g. confidence intervals)
☐	☒	For null hypothesis testing, the test statistic (e.g. *F*, *t*, *r*) with confidence intervals, effect sizes, degrees of freedom and *P* value noted<br>*Give P values as exact values whenever suitable.*
☒	☐	For Bayesian analysis, information on the choice of priors and Markov chain Monte Carlo settings
☒	☐	For hierarchical and complex designs, identification of the appropriate level for tests and full reporting of outcomes
☐	☒	Estimates of effect sizes (e.g. Cohen's *d*, Pearson's *r*), indicating how they were calculated

Our web collection on statistics for biologists contains articles on many of the points above.

Software and code

Policy information about availability of computer code

Data collection	No software was used.
Data analysis	All custom code used for this study is available in the linked github repository https://github.com/walsh-quail-labs/IMC-Lung. Code for IMC cell segmentation was written in Matlab v2019b and Python (version 3.7.12). All deep learning analysis steps were performed in Python (version 3.7.12) and the TensorFlow (version 2.8.0) framework alongside Keras (2.11.0), RandomOverSampler (Version: 0.9.1) and Scikit-learn (version 1.0.2). Prism 9.1.0 (GraphPad) and RStudio version 4.2.2 were used for statistical analyses.

For manuscripts utilizing custom algorithms or software that are central to the research but not yet described in published literature, software must be made available to editors and reviewers. We strongly encourage code deposition in a community repository (e.g. GitHub). See the Nature Portfolio guidelines for submitting code & software for further information.

Data

Policy information about availability of data

All manuscripts must include a data availability statement. This statement should provide the following information, where applicable:

- Accession codes, unique identifiers, or web links for publicly available datasets
- A description of any restrictions on data availability
- For clinical datasets or third party data, please ensure that the statement adheres to our policy

The source data supporting findings in this study, including high-dimensional TIFF images, and associated clinical data have been deposited at https://doi.org/10.5281/zenodo.7383627. Raw primary imaging data can be obtained from the authors directly upon reasonable request.

Human research participants

Policy information about studies involving human research participants and Sex and Gender in Research.

Reporting on sex and gender	Lung cancer is the leading cause of cancer death for both males and females. Our study has equal representation of both male and female patient samples. Additionally, sex as a biological variable was accounted for in our analysis and subsequent correlations (Figure 2a-b; Extended Figure 3a,d; Extended Figure 5c; Extended Figure 8a). Our study examined fundamental biological variables without consideration of socio-cultural factors so gender was not considered.
Population characteristics	Population characteristics can be found in Supplementary Table 1 & 12. We obtained treatment-naive biobanked surgical resection and biopsy lung adenocarcinoma tumor specimens. All biobanked primary lung tumor surgical specimens and biopsies of patients that had an assessment of the primary histological subtype of their tumor as well as appropriate consent were included.
Recruitment	Primary lung adenocarcinoma patients that underwent surgical resection or biopsy at Laval University from February 1996 till July 2020 were retrospectively contacted to sign a consent form for research through the IUCPQ institutional biobank. All participants who agreed to the consent form were recruited. This cohort was enriched for early-stage patients, therefore our findings may be biased towards these groups. Almost half of lung cancer patients are diagnosed at stage IV. The enrichment for early-stage patients in our cohort reflects the inability to collect surgical resection samples from primary lung adenocarcinoma of advanced stage as surgery is not an available treatment modality for these patients. For the validation cohort, stage 1 primary lung adenocarcinoma patients that underwent surgical resection or biopsy at the McGill University Health Centre between February 2012 and May 2022 were retrospectively contacted to sign a consent form for research.
Ethics oversight	The protocol for human sample biobanking was approved (ethics, scientific and final) by the IUCPQ, Protocol Number: IRB #2022-3474, 22090. For the McGill University Health Centre cohort, the protocol was approved with IRB # 2014-1119 and 2019-5253.

Note that full information on the approval of the study protocol must also be provided in the manuscript.

Field-specific reporting

Please select the one below that is the best fit for your research. If you are not sure, read the appropriate sections before making your selection.

☒ Life sciences ☐ Behavioural & social sciences ☐ Ecological, evolutionary & environmental sciences

For a reference copy of the document with all sections, see nature.com/documents/nr-reporting-summary-flat.pdf

Life sciences study design

All studies must disclose on these points even when the disclosure is negative.

Sample size	For our study, 416 primary lung adenocarcinomas were included. A second cohort containing 120 images from 60 stage 1 patients was also analyzed as a validation cohort. We included all samples we had access to and as such sample size was not specified.
Data exclusions	All patients were included in this study.
Replication	The k-fold cross validation method was used, where the data was split into 5 folds, with 20% of the data for each fold. In our experiments, we considered 4 of the folds (80% of the patients) as the training data and the remaining fold (20%) for testing to evaluate the prediction accuracy. An independent dataset was used as the validation cohort.
Randomization	Patients were randomly assigned to each fold for the k-fold cross validation methods for both the training and testing data.
Blinding	TMA construction, slide staining and image acquisition were all blinded to clinical and pathological data.

Reporting for specific materials, systems and methods

We require information from authors about some types of materials, experimental systems and methods used in many studies. Here, indicate whether each material, system or method listed is relevant to your study. If you are not sure if a list item applies to your research, read the appropriate section before selecting a response.

Materials & experimental systems

n/a	Involved in the study
☐	☒ Antibodies
☒	☐ Eukaryotic cell lines
☒	☐ Palaeontology and archaeology
☒	☐ Animals and other organisms
☒	☐ Clinical data
☒	☐ Dual use research of concern

Methods

n/a	Involved in the study
☒	☐ ChIP-seq
☒	☐ Flow cytometry
☒	☐ MRI-based neuroimaging

Antibodies

Antibodies used	All antibodies used in this study were titrated for each lot. Information about all antibodies can be found in Supplementary Table 2.
Validation	All antibodies were tested on multiple control tissues including spleen, tonsil, placenta, appendix, thymus, lung and tumor tissue for accuracy and cell type specificity.
